# Developing an accurate model of spot-scanning treatment delivery time and sequence for a compact superconducting synchrocyclotron proton therapy system

**DOI:** 10.1186/s13014-022-02055-w

**Published:** 2022-05-07

**Authors:** Lewei Zhao, Gang Liu, Shupeng Chen, Jiajian Shen, Weili Zheng, An Qin, Di Yan, Xiaoqiang Li, Xuanfeng Ding

**Affiliations:** 1 Department of Radiation Oncology, Beaumont Health System, Royal Oak, MI 48073 USA; 2 Cancer Center, Union Hospital, Tongji Medical College, Huazhong University of Science and Technology, Wuhan, People’s Republic of China; 3 Department of Radiation Oncology, Mayo Clinic Arizona, Phoenix, AZ 85054 USA

**Keywords:** Proton beam, Time prediction, Burst switching

## Abstract

**Background:**

A new compact superconducting synchrocyclotron single-room proton solution delivers pulsed proton beams to each spot through several irradiation bursts calculated by an iterative layer delivery algorithm. Such a mechanism results in a new beam parameter, burst switching time (BST) in the total beam delivery time (BDT) which has never been studied before. In this study, we propose an experimental approach to build an accurate BDT and sequence prediction model for this new proton solution.

**Methods:**

Test fields and clinical treatment plans were used to investigate each beam delivery parameter that impacted BDT. The machine delivery log files were retrospectively analyzed to quantitatively model energy layer switching time (ELST), spot switching time (SSWT), spot spill time (SSPT), and BST. A total of 102 clinical IMPT treatment fields’ log files were processed to validate the accuracy of the BDT prediction model in comparison with the result from the current commercial system. Interplay effect is also investigated as a clinical application by comparing this new delivery system model with a conventional cyclotron accelerator model.

**Results:**

The study finds that BST depends on the amount of data to be transmitted between two sequential radiation bursts, including a machine irradiation log file of the previous burst and a command file to instruct the proton system to deliver the next burst. The 102 clinical treatment fields showed that the accuracy of each component of the BDT matches well between machine log files and BDT prediction model. More specifically, the difference of ELST, SSWT, SSPT, and BST were (− 3.1 ± 5.7)%, (5.9 ± 3.9)%, (2.6 ± 8.7)%, and (− 2.3 ± 5.3)%, respectively. The average total BDT was about (2.1 ± 3.0)% difference compared to the treatment log files, which was significantly improved from the current commercial proton system prediction (58 ± 15)%. Compared to the conventional cyclotron system, the burst technique from synchrocyclotron effectively reduced the interplay effect in mobile tumor treatment.

**Conclusion:**

An accurate BDT and sequence prediction model was established for this new clinical compact superconducting synchrocyclotron single-room proton solution. Its application could help users of similar facilities better assess the interplay effect and estimate daily patient treatment throughput.

**Supplementary Information:**

The online version contains supplementary material available at 10.1186/s13014-022-02055-w.

## Background

The pencil beam scanning (PBS) technique has become a popular treatment modality in the field of proton beam therapy [1]. Compared to passive-scattering proton therapy, this technique offers a conformal dose distribution to target and better normal tissue sparing capability [2, 3]. However, PBS technique is sensitive to the interplay effect between proton spot delivery sequence and respiratory motion. This phenomenon leads to under- and overdoses in some lung and liver cancer cases [4, 5]. 4D dose calculation methods (such as the 4D dynamic dose method) are often used to assess the breathing-induced interplay effect by synchronizing the patient-specific breathing pattern with the proton machine spot delivery sequence [6, 7]. For 4D dose calculation, it is important to understand the structure of the beam and beam delivery sequence. Thus, a proton system’s beam delivery time (BDT) prediction model plays a key factor for an accurate estimation of the interplay effect through the 4D dynamic dose calculation and simulation. In addition, the beam time of a proton therapy center is invaluable. An accurate prediction of the treatment BDT would help estimate the daily patient throughput, which is critical to the clinical operation of a proton therapy center [8].

With the surge of demands in proton beam therapy worldwide, there is an immediate need to lower the investment cost of a new proton therapy center [9]. Recent interviews with vendors and experts showed that the trend in proton therapy has swung towards smaller, compact, single-room proton therapy system installations and moved away from traditional large, multi-room treatment centers around the world [10]. In the last decades, new gantries and accelerator systems with a much smaller footprint were introduced into the market [11]. These compact systems can fit into an existing facility or a smaller landscape which is especially important for hospitals within the city limits [12]. One of the popular proton therapy systems is the IBA ProteusONE® which consists of a compact superconducting synchrocyclotron accelerator (S2C2) [13]. Since its debut in 2014 [14], this new proton therapy system has been adopted by more than 20 institutions [15]. This new accelerator, S2C2, not only extracts a unique high intensity pulsed beam but also delivers every spot in each energy layer through several radiation bursts [16]. Such a mechanism results in a new beam parameter: burst switching time (BST) which is new to the particle therapy community. Meanwhile, the current IBA ProteusONE®’s treatment console (also called Scanning Controller Algorithm, ScanAlgo) could not predict an accurate BDT (see Additional file 1: Fig. s1). Thus, it was not clinically useful at this moment.

Several efforts have been made to address the BDT model based on the multi-room proton centers equipped with a traditional accelerator such as HITACHI ProBeat [17], a synchrotron accelerator system, and IBA ProteusPLUS®, a cyclotron accelerator system [18]. To the best of our knowledge, there is no published beam delivery sequence and BDT model for IBA ProteusONE®, a popular but new proton compact superconducting synchrocyclotron accelerator system yet. Thus, it limits the clinical users’ ability to assess the interplay effect for moving targets as well as the patient treatment throughput estimation and scheduling for a proton center using this system. This study proposes an experimental approach to derive each component of the BDT for this new superconducting synchrocyclotron proton therapy system. These derived parameters were used to model the BDT and its proton beam delivery sequence. Then, this BDT prediction model was validated using 102 clinical treatment IMPT fields by comparison with the machine log file and current prediction results from IBA ProteusONE®’s treatment console. The impact of the machine-specific model on the estimation of interplay effect is investigated. The experimental approach could be adopted by other proton institutions to model their system and the model will be useful in the precise interplay effect evaluation.

## Methods

Before introducing our methods, first, we need to describe specific characteristics of IBA ProteusONE®, which is featured by an iterative layer delivery algorithm.

## A: IBA ProteusONE® beam delivery sequence and iterative layer delivery algorithm

**Fig. 1:**
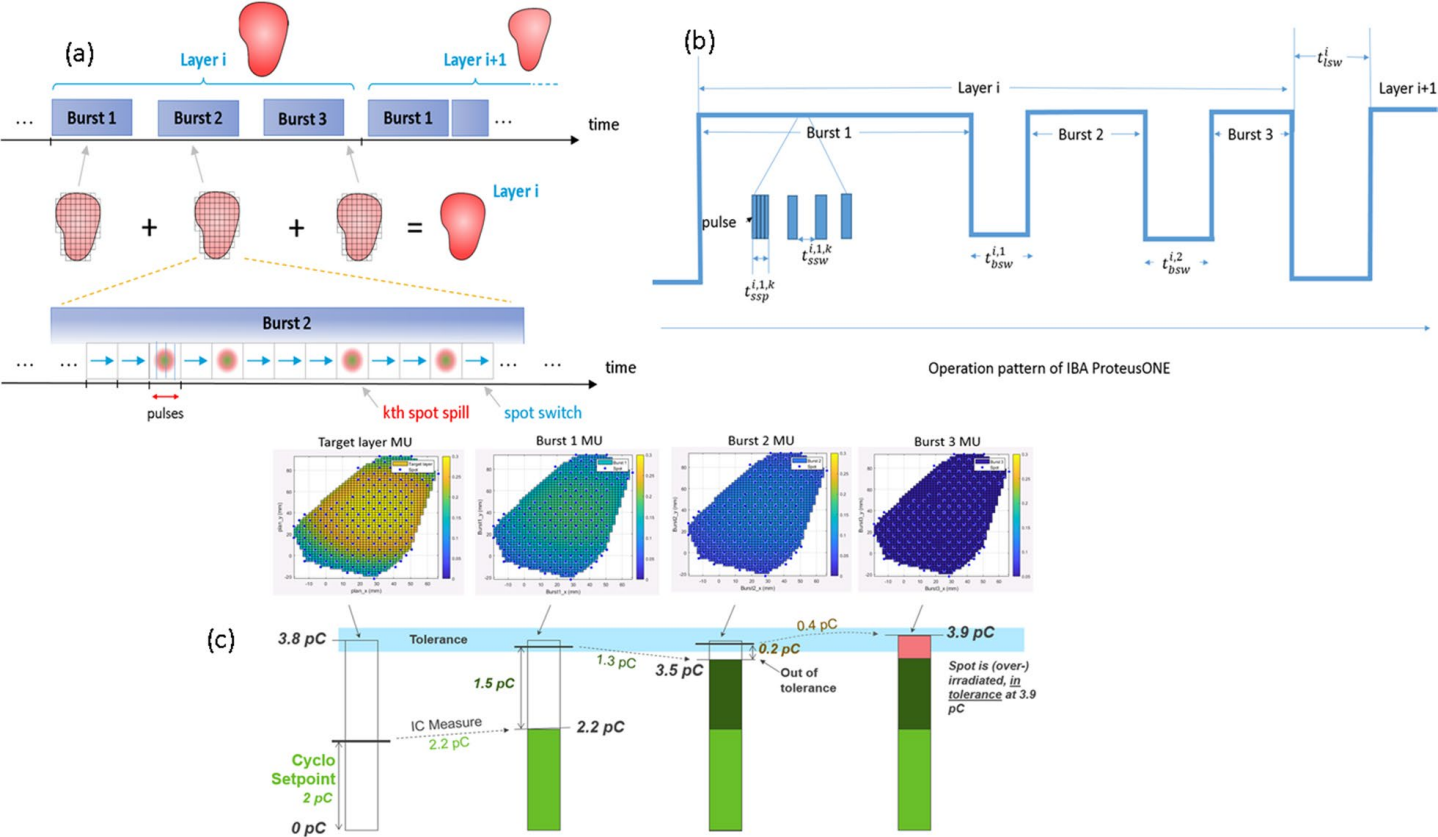
A schematic of the IBA ProteusONE**®** delivery sequence. **a** The pattern delivers through three radiation bursts per energy layer [17]. **b** The filled rectangles are the spot spill time $t_{ssp}^{i,j,k}$, and the gaps between them are the spot switching time $t_{ssw}^{i,j,k}$. The gap between consecutive bursts represents burst switching time $t_{bsw}^{i,j}$ and the gap between layers represents layer switching time $t_{lsw}^{i}$. **c** An example of the IBA’s iterative layer delivery algorithm. This algorithm divides each energy layer normally in three radiation bursts such that the error in total charge delivered to each spot will not exceed the clinical tolerance [17]. The Monitor Unit (MU) distribution is displayed using a lung case as an example, with energy layer 156.2 MeV and 178 spots from a treatment field

IBA ProteusONE® is a compact single room proton therapy solution equipped with a 220-degree compact gantry, a compact superconducting synchrocyclotron (S2C2) with a dedicated PBS nozzle for discrete spot scanning. This S2C2 delivers a high-intensity pulsed proton beam with a 1 kHz frequency while the duration of beam application within one pulse is 7 µs [19]. It is an engineering challenge to ensure both beam stability and dose delivery efficiency simultaneously. In traditional continuous beam current scanning, the radiation delivery sequence is spot by spot and layer by layer. As opposed to the continuous beam current extracted from an isochronous cyclotron, the time to deliver a pulse is too short for an effective feedback loop during the pulse delivery itself. Delivering a spot with a single pulse can therefore be subject to unacceptable uncertainty. A detailed example is specifically illustrated in the Additional file 1 (10% is only given as an example in our paper. The actual uncertainties may be higher or lower depending on synchrocyclotron status and parameters, such as VDee, see in the Additional file 1: Fig. s2). For example, cyclotron setpoint could be 2pC, but the system may extract 2.2pC (Fig. 1c). To achieve the clinical treatment delivery accuracy, an iterative layer delivery algorithm was introduced and applied to IBA ProteusONE® [16] (Fig. 1). It divides each spot MU usually into three bursts, like an adaptive layer repainting technique via a feedback loop based on the previous burst [16]. In other words, the fluence in each burst is inhomogeneous compared to the traditional layer repainting technique [20, 21], which delivers the spot MU homogeneously. A schematic of the IBA ProteusONE® delivery sequence is described in Fig. 1. An intuitive example of the spot delivery sequence based on the iterative layer delivery algorithm is included in the Additional file (Fig. 1c and the specific illustration in the Additional file 1).

## B: Beam delivery system parameters and modeling

With the discrete spot scanning technique in IBA ProteusONE®, the proton treatment plan is delivered “spot-by-spot”, “burst-by-burst” and “layer-by-layer” (Based on this machine-specific model, in each burst, all spots within an energy layer are being irradiated, see 3.C.3b for how the particle number is controlled for each spot). The total BDT ($$T_{BDT}$$) was composed of total energy layer switching time ($$T_{LSW} )$$, total burst switching time ($$T_{BSW}$$), total spot switching time ($$T_{SSW}$$) and the total spot spill time ($$T_{SSP}$$). The model to predict $$T_{BDT}$$ can therefore be written as:

$$T_{BDT} = T_{LSW} + T_{BSW} + T_{SSW} + T_{SSP}$$

$$T_{LSW} = \mathop \sum \limits_{i = 1}^{{N_{layer} - 1}} t_{lsw}^{i}$$

$$T_{BSW} = \mathop \sum \limits_{i = 1}^{{N_{layer} }} \mathop \sum \limits_{j = 1}^{{N_{burst}^{i} - 1}} t_{bsw}^{i,j}$$

$$T_{SSW} = \mathop \sum \limits_{i = 1}^{{N_{layer} }} \mathop \sum \limits_{j = 1}^{{N_{burst}^{i} }} \mathop \sum \limits_{k = 1}^{{N_{spot}^{i,j} - 1}} t_{ssw}^{i,j,k}$$

$$T_{SSP} = \mathop \sum \limits_{i = 1}^{{N_{layer} }} \mathop \sum \limits_{j = 1}^{{N_{burst}^{i} }} \mathop \sum \limits_{k = 1}^{{N_{spot}^{i,j} }} \left( {n_{pulse}^{i,j,k} \times 1ms} \right)$$

where the symbol *t* is defined as the time for single layer (superscript *i*), single burst (subscript *j*) and single spot (subscript *k*); $N_{layer}$ is the total number of layers in the beam, $N_{burst}^{i}$ is the total number of bursts in the layer *i* ( for most layers, $N_{burst}^{i} = 3$), $N_{spot}^{i,j}$ is the total number of spots in the layer *i*, burst *j*, $n_{pulse}^{i,j,k}$ is the total number of pulses in ith energy layer, jth burst and kth spot.

To build an accurate delivery sequence and BDT model, we proposed a method to experimentally measure the machine irradiation intensity of the pulse, which is to solve the $n_{pulse}^{i,j,k}$(Sect. 2.C.3) and derive the BST or $T_{BSW}$ (Sect. 2.C.4) along with the other standard beam parameters such as $T_{LSW}$ (Sect. 2.C.1), $T_{SSW}$ (Sect. 2.C.2) and $T_{SSP}$(Sect. 2.C.3).

## C: Generation of test fields

Each test field was created in a commercial treatment planning system (RaySearch Laboratories AB, Stockholm, Sweden, RayStation ver. 6.) with a different spot pattern and beam characteristics such as different Monitor Unit (MU) weighting, energy layer switching sequence, spot positions etc. These test fields were delivered in the clinical mode where the machine log file records each radiation burst in terms of the charges per pulse, number of pulses ($n_{pulse}^{i,j,k}$) and spot positions with 1 ms sampling frequency. Then, the log files were retrospectively analyzed to derive each component of BDT of the proton system, such as energy layer switching time (ELST), spot scanning speed in x, y and diagonal direction, burst switching time (BST), and spot spill time (SSPT). The total BDT from the log file was manually validated using a stopwatch.

## C.1: Experiment to determine ELST

The total ELST is the sum of switching time from the sequential energy layers:$T_{LSW} = \mathop \sum \limits_{i = 1}^{{N_{layer} - 1}} t_{lsw}^{i}$. The S2C2 accelerator extracts the radiation pulses at the maximum energy of around 227 MeV. The energy layer selection system is very similar to a traditional cyclotron system which is based on a physical wedge and the related beamline and magnetic field configurations [22]. The ELST may depend on the energy switching interval or energy switching directions (e.g., descending or ascending) which is impacted by the magnetic hysteresis [23]. To quantitatively determine the ELST in different scenarios, a series of test fields with different energy layers switching intervals were created in both descending and ascending orders (see Additional file 1: Table s1). Each energy layer incorporated a single spot directed at the isocenter (i.e. x = 0, y = 0) with 0.02 MU weighting (MU_min_ = 0.01 in IBA ProteusONE®), the relationship of ion numbers per MU is attached in the Additional file 1: Fig. s3. By using only one spot and a small ion number, this approach eliminated spot switching time (i.e $T_{SSW} = 0 {\text{s}}$) and minimized the spot spill time ($T_{SSP}$) to within several milliseconds, which is negligible compared to the ELST (varies from 0.7 s to 6 s). Therefore, the ELST can be obtained directly from the log files recorded during the experiment.

## C.2: Spot scanning speed and switching time

The total spot switching time (SSWT) is the sum of switching time between the sequential spots:$T_{SSW} = \mathop \sum \limits_{i = 1}^{{N_{layer} }} \mathop \sum \limits_{j = 1}^{{N_{burst}^{i} }} \mathop \sum \limits_{k = 1}^{{N_{spot}^{i,j} - 1}} t_{ssw}^{i,j,k}$. Each SSWT includes two components (1) scanning magnet (x,y) preparation “dead time”, and (2) the spot scanning time required to steer the spot from a previous position to a new position. In order to isolate and derive the scanning magnet (x,y) preparation “dead time” between the spots without steering the magnet, a test field was created with two energy layers (200 MeV and 150 MeV), 10 spots per layer, all spots directed to isocenter, and 0.02 MU/spot similar to Shen et al.’s method [17]. The SSWT is then equivalent to the “dead time” between consecutive spots. In other words, the “dead time” is the SSWT when the switching distance is zero. Once we know the scanning magnet preparation “dead time”, we are able to derive the spot scanning time and speed later.

**Fig. 2:**
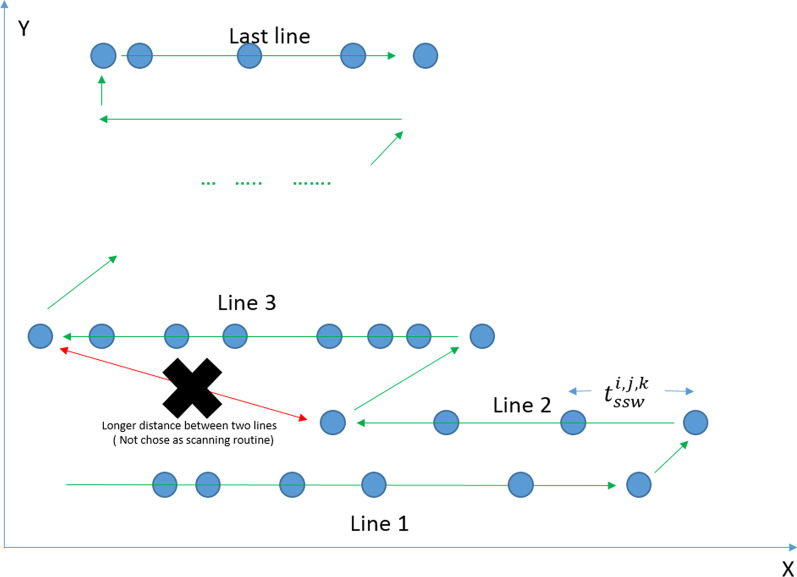
The IBA ProteusONE® spot scanning sequence. The IBA ProteusONE® controller scanning proton beam delivers spot by spot, line by line and chooses the shorter distance during the line switching

To derive the accurate spot scanning time model, we need first to understand the IBA ProteusONE® spot scanning sequence or scanning controller. IBA ProteusONE® controller scans the spot starting from the left lower corner (from the Beam Eye View) of the treatment field spot by spot, line by line, and all the way to the top. During the line switching, it chooses the route from the last spot position of the previous line to the initial spot (left or right end) of the next line whichever is shorter (See Fig. 2). Based on this prior knowledge, we are able to derive the spot scanning speed or magnetic scanning speed.

To derive the magnetic scanning speed in x-direction (spot positions with the same y-coordinate), ten clinical IMPT cases’ treatment log files were used. These ten cases cover varieties of spot x scanning distances and sequences in a clinical setting. However, spot scanning rarely moves strictly along the y-direction in clinical cases due to the hexagon spot placement setting in TPS. (First of all, x-scanning direction is faster than y-direction. The IBA proton system delivers the spot in x-direction and then switches the row in y-direction. Secondarily, the clinical plans use hexagon spot placement, as a result, spots are unlikely to scan along y-direction only in clinical cases.). To quantitatively model the y-direction scanning speed(spot positions with the same x-coordinate), we designed the test fields that the spot with minimum 0.02 MU travels in y-direction only ($\left| {x_{i,j,k + 1} - x_{i,j,k} } \right| = 0)$ at thirty-four different y-distance interval $\left| {y_{i,j,k + 1} - y_{i,j,k} } \right| = 1,2,3, \ldots ,7,8,9, 10 + 5n \left( {n = 0,1,2,..16} \right), 107, 119, 155, 175, 176, 177, 180, 225$(mm). We investigated these sets of treatment plans several times, while for each set of plans the absolute x-coordinate was altered to investigate whether the x-position has an influence on the SSWT in y-direction. In most clinical cases where hexagonal spot placement grid was used in RayStation TPS, line switching uses diagonal direction. In other words, the last spot of the previous line and the first spot of the next line normally do not share the same x coordinate because of the hexagonal spot grid. To test diagonal line switching speed (spot switching in both x and y coordinates), we designed a series of rectangular diagonal test fields so that the spot traveled in different x, y diagonal directions and distances during the line switching. Details of those rectangular-shaped test fields were listed in the Additional file 1: Table s2. The y-direction and diagonal experiments were repeated for 70, 100, 150, 200, and 220 MeV.

## C.3: Spot spill time

The total spot spill time (SSPT) is the total number of pulses in unit millisecond: $T_{SSP} = \mathop \sum \limits_{i = 1}^{{N_{layer} }} \mathop \sum \limits_{j = 1}^{{N_{burst}^{i} }} \mathop \sum \limits_{k = 1}^{{N_{spot}^{i,j} }} \left( {n_{pulse}^{i,j,k} \times 1\;{\text{ms}}} \right)$. This synchrocyclotron accelerator, S2C2, delivers one or more pulses to each spot during the radiation burst. To solve the SSPT of the IBA ProteusONE®, we will first need to know the intensity of the pulse or charges/MU per pulse (we use the notation: *charges per pulse* in this paper as a simplification purpose). Second, we will need to know the estimated amount of pulses per spot which is going to be delivered during each radiation burst.

## C.3a charges per pulse

The charges per pulse are controlled by the voltage between the Dees (V_Dee_) of the S2C2. A higher V_Dee_ is associated with a larger amount of the charges per pulse (the relationship of charge per pulse and V_Dee_ is attached in the Additional file 1: Fig. s4). However, the charges per pulse have certain statistical uncertainties (see look-up table in the Additional file 1: Fig. s2), which require multiple radiation bursts to achieve the clinical treatment delivery accuracy [13]. In a normal clinical setting, the system utilizes the minimum number of pulses to finish the treatment delivery. In other words, it uses the charges per pulse close to the maximum efficiency (the amount of charges per pulse at 100% of $V_{Dee}$ setting) to irradiate each spot in large MU. To fill the remaining charges of the spot in a very small MU weighting (much less than the maximum charges in a pulse, or maximum efficiency), the system adjusts V_Dee_ to a smaller value (e.g. V_Dee_ setting is around 75%). So it can hit the target (the total MU/charges of the spot in the energy layer in the treatment plan) within a clinically acceptable accuracy in a third radiation burst (Fig. 1) because the statistical variation of pulse charges in the last burst is relatively small compared to the target. As there are statistical uncertainties in the charges per pulse, which were accelerated and extracted from the S2C2, it would be difficult to directly model or predict the exact amount of charge per pulse used in each radiation burst. For a practical and simplification purpose, we introduce a maximum efficiency ($v_{i}$) irradiation model in calculating the SSPT, assuming the maximum charges per pulse at V_Dee_ = 100% are used without statistical variations. In this model, it is critical to know the machine beamline’s maximum efficiency or max charges per pulse from 70 to 227 MeV. In this section, the maximum charges of pulse were measured directly by adjusting the V_Dee_ to 100% at different energies from 70 to 227 MeV.

## C.3b Determine the number of the pulse to be delivered for the spot in each radiation burst

**Fig. 3:**
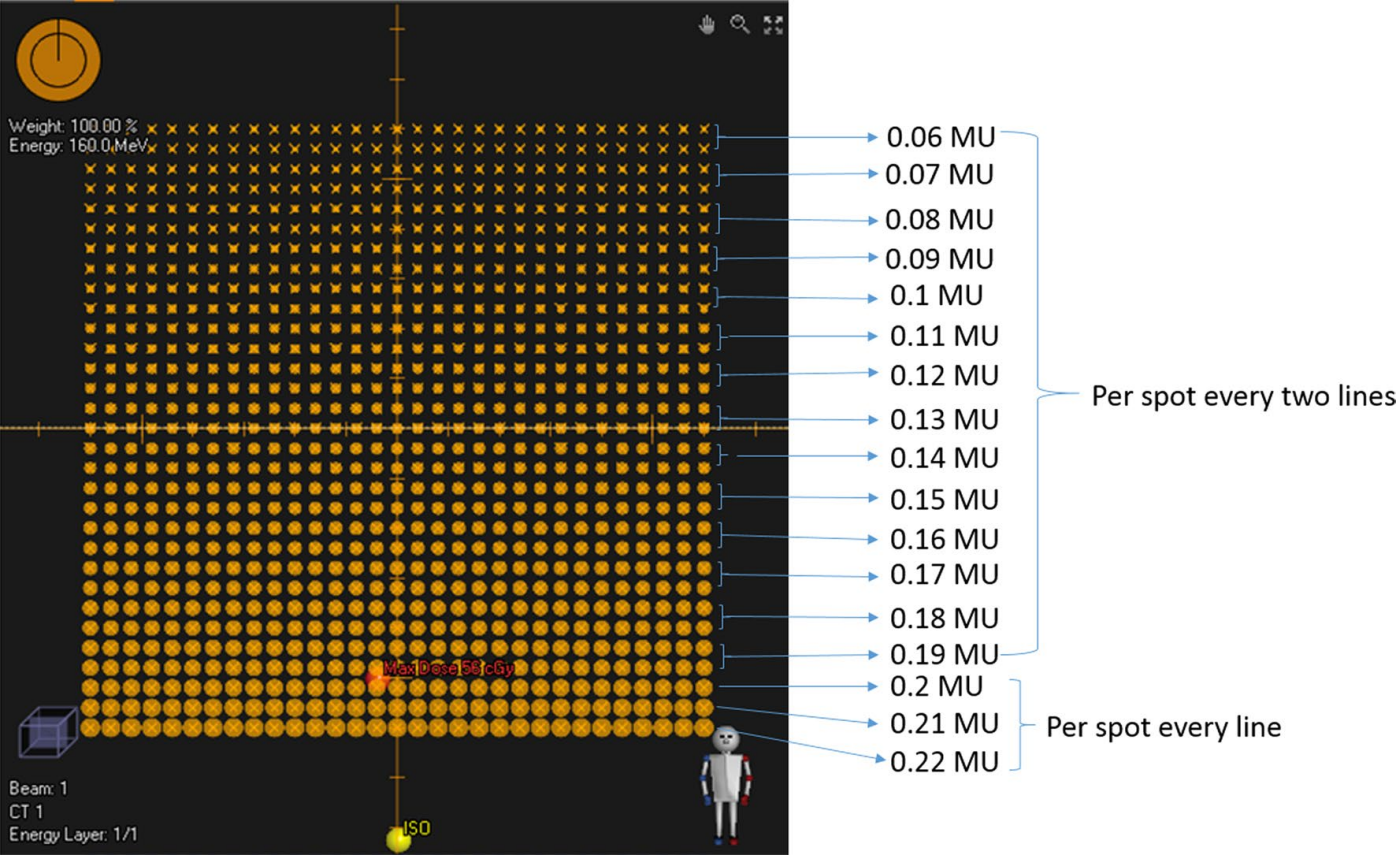
Spot spill time experiment design. One of test fields pattern used to measure the 0.06–0.22 MU weighting per radiation burst and estimate the number of pulse in RayStation 6 (see Additional file 1: Table s3). Each line spots has the same MU. From the bottom to above, the first 3 lines have different MU. For other lines, spot MU changed by every two lines

The IBA ProteusONE® system clinical mode normally takes three radiation bursts to reach the target (total charge or MU of each spot) in an energy layer. In other words, the spot in each energy layer usually will be painted three times through the iterative layer delivery mechanism. Since the accelerator and beamline statistical stability and uncertainties parameters vary slightly daily and this information is not accessible by the clinical user, it is almost impossible to predict an exact amount of charges or MU weighting to be delivered in each radiation burst. The measurements were based on many tests and experiments in different days that could also smooth out the daily variations during the modeling. In this study, we averaged the relative MU weighting from each radiation burst throughout the range of energy layers as well as various gantry angles. This information was used to estimate the number of pulses in each radiation burst. Thus, a series of square test fields (Fig. 3) were created with 17 different energies ranging from 70 to 227 MeV, and MU per spot ranged from 0.015 MU to 15 MU. These test fields were delivered at gantry angles 0, 45, 90, 135, and 180 degrees (see Additional file 1: Table s3).

## C.4: Burst switching time

The total burst switching time (BST) is the sum of switching time between the sequential radiation bursts: $T_{BSW} = \mathop \sum \limits_{i = 1}^{{N_{layer} }} \mathop \sum \limits_{j = 1}^{{N_{burst}^{i} - 1}} t_{bsw}^{i,j}$. During a radiation burst, each spot is irradiated using one or more pulses. Based on the charges which were delivered to the spot in the previous radiation burst, the iterative layer delivery algorithm calculates the next radiation burst. Therefore, the proton system controller needs to receive the previous radiation burst information before calculating the following radiation burst. So we hypothesize that this new beam delivery time parameter, BST, is directly related to the size of data or file which is transferred between the controller and delivery system, including network transmission speed, file and data size, network response time, and controller calculation speed. Typically, the network latency or network response time remains the same. Two types of files exchange information between the controller and proton beam delivery system: an irradiation record log file from the previous radiation burst and a command file to instruct the proton system to deliver the next radiation burst (see Additional file 1: Fig. s5). Typically, the file size can be 1–3500 kB for the irradiation record log file and 1–1400 kB for the command file.

To derive which beam parameters lead to file sizes and related network transmission speed, we will first need to know the structure of these log files and command files in regard to the recording time and other machine beam parameters such as spot and pulse information. The log files and command files of the same ten clinical cases (Sect. 2.C.2 x-direction spot switching time test) are used in this section. As the proton system records the radiation delivery information row by row at 1 kHz (see Additional file 1: Fig. s6), machine treatment log file size reflects the irradiation number of the radiation burst ($$N_{SSW}^{i,j} + N_{pulse}^{i,j}$$), where $$N_{SSW}^{i,j} = T_{SSW}^{i,j} /1ms$$ is the total spot switching number in this burst. The command file contains spot information and the number of pulses to be delivered in each spot. Each of this information occupies one row of the csv file (see Additional file 1: Fig. s7). Therefore, the command file size depends on the number of pulses and spots to be delivered in the following burst ($$N_{pulse}^{i,j + 1} + N_{spot}^{i,j + 1}$$). Based on this information, we are able to plot the BST as a function of the transmission data summation between the two radiation bursts ($$N_{SSW}^{i,j} + N_{pulse}^{i,j} + N_{pulse}^{i,j + 1} + N_{spot}^{i,j + 1} )$$. The “dead time”, which includes network response time and controller calculation time, can be estimated by using the vertical intercept of the linear fitting. In summary, BST prediction model can be established based on the four parameters: the total spot switching number of the previous burst ($$N_{SSW}^{i,j}$$), the total number of pulses in the previous burst ($$N_{pulse}^{i,j}$$), the total number of pulses in the following burst ($$N_{pulse}^{i,j + 1}$$) and the total number of spots in the following burst ($$N_{spot}^{i,j + 1}$$) and a constant time or “dead time”.

## D Validation of Beam Delivery Time (BDT) model using clinical treatment fields

The BDT prediction model was verified through 102 IMPT clinical treatment fields. Each BDT component including ($T_{LSW}$, $T_{SSW}$, $T_{SSP} ,{ }T_{BSW}$) were compared to the machine log files. The recorded total beam delivery time was obtained by subtracting the start irradiation time from the end irradiation time. Also, the model's overall accuracy predicted time and vendor predicted time were compared with log files.

## E Interplay effects evaluation

To test whether or not machine-specific delivery time and sequence’s concept is important to interplay effect evaluation, the in-house IBA ProteusONE® model developed in this study was compared with a simplified proton system model with non-burst mechanism based on the IBA ProtuesPLUS® model, a conventional cyclotron accelerator system, which was published by West German Proton Therapy Center in Essen [18] (WPE). A digital thoracic 4DCT phantom image set [24] with a rigid target (radius 2 cm) in the right lung was used for the study. The 4DCT dataset has 10 phase images. Target motion [24] was simulated based on the periodic respiratory motion with an amplitude of 5 mm. The breathing cycle is 4 s. A two-field Single Field Uniform Dose (SFUD, which is referred to as the optimization technique in which one field covers the entire targets [25].) plan was generated using PA and lateral fields [26] (In this case, two-field SFUD plan means each field cover the entire target with 50% of the prescription dose, see Additional file 1: Fig. s8). Spot delivery sequence was compared among the WPE model, machine-specific P1 model and machine logfile. To access the interplay effect based on the two delivery system models. 4D dynamic dose accumulation method [24] for ten different starting phases was used, assuming the breathing pattern remains the same. To calculate a single fraction 4D dynamic dose, the dose calculated on each phase image was accumulated via the deformable image registration to the reference phase (phase 50%). D99 (Dose received by 99% of target volume) of the target is used to estimate the target coverage.

As we mentioned in 2.A, the iterative layer delivery algorithm of the IBA ProteusONE® system plays a role like an adaptive layer repainting technique. The three-burst mechanism is similar to a three-layer repainting delivery sequence (the number of applied MU for each burst will be stated in 3.C.3b). But spot MU delivered in each repainting is different for the three-burst mechanism. More details were explained in Sect. 3.C.3b.

Different delivery techniques such as standard delivery (volumetric repainting number $N_{vol}$ = 1) and volumetric repainting delivery ($N_{vol}$ = 2–4) were simulated based on the machine-specific ProteusONE® delivery sequence model, a burst mechanism, derived from this study and compared to the WPE model, a non-burst mechanism. DVH (dose-volume histogram) band curves were plotted. In particular, target coverage D99 (dose received by 99% of volume) was analyzed.

## Results

The total BDT and other beam parameters were independently imported from the log file with a modified MATLAB package based on the OpenReggui platform [27].

## A: Energy layer switching time modeling

**Fig. 4:**
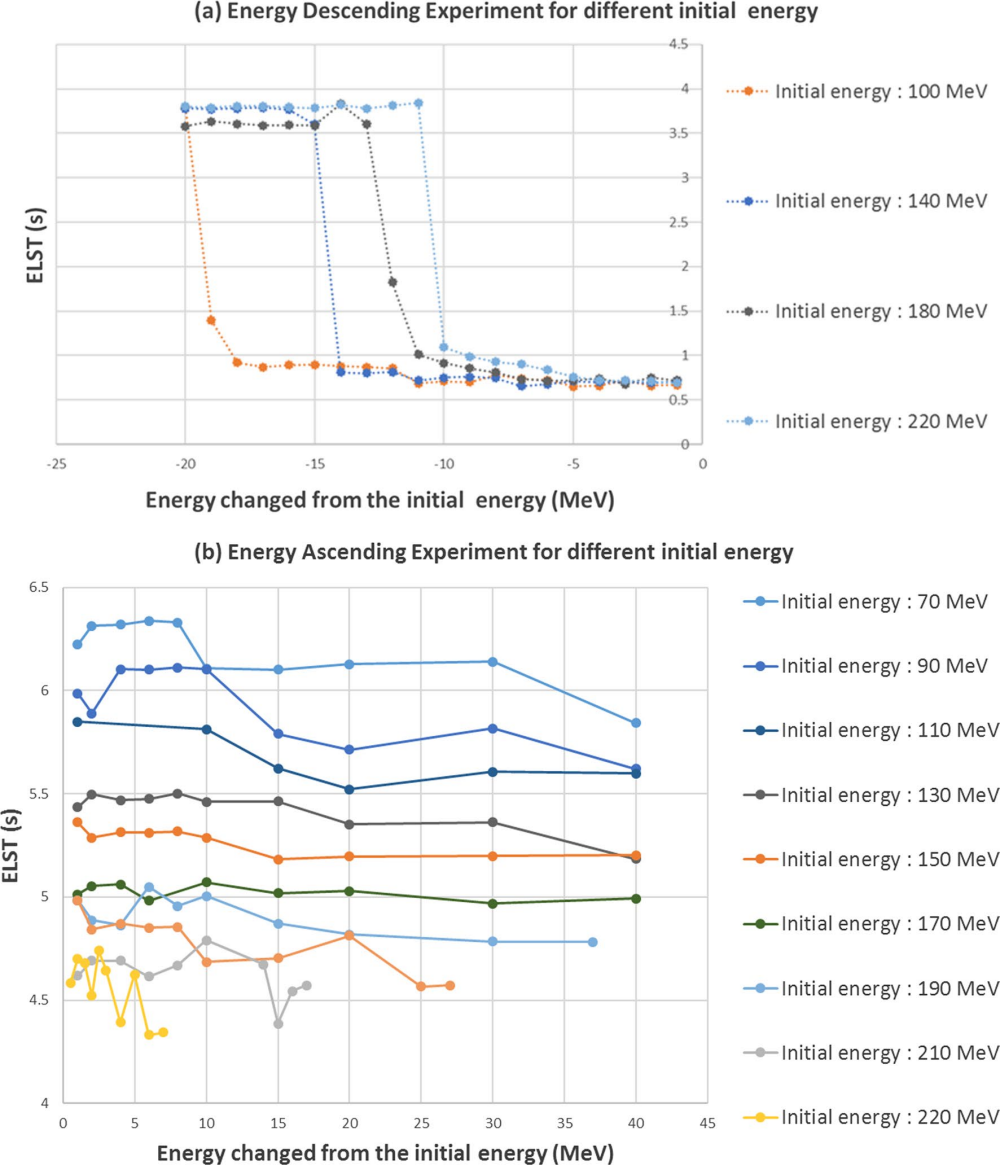
Energy layer switching time experiment result. **a** Energy layer descending switching time as a function of the energy switching interval or called energy layer spacing (Initial energy from 100 to 220 MeV). **b** Energy layer ascending switching time is constant in contrast to the descending energy selection

The energy descending switching is the current clinical IMPT standard operation [28]. Energy layer switching time was plotted as a function of energy switching interval in a descending energy layer switching sequence (Fig. 4a). The ELST varies from 0.6 to 0.8 s (average 0.7 s) in most scenarios when the energy difference between two consecutive layers is less than 10 MeV. However, the ELST increases significantly when the decreased energy separation interval is greater than 10 MeV depending on the initial energy layers. The ELST for ascending energy is relatively stable between 5 and 6 s. For simplification, we use 5.5 s as a constant number in our ELST model ascending case (see Fig. 4b).

Thus, the ELST (s) can be modeled as a step function depending on the energy changed from the initial energy layer ($E_{i}$):

$$t_{lsw}^{i} = \left\{ {\begin{array}{*{20}l} {3.75} \hfill & {if\;\Delta E_{i} < S\left( {E_{i} } \right)} \hfill \\ {0.7} \hfill & {if S\left( {E_{i} } \right) \le \;\Delta E_{i} < 0} \hfill \\ {5.5} \hfill & {if \;\Delta E_{i} > 0} \hfill \\ \end{array} } \right.\; \left( {{\text{unit}}:{\text{s}}} \right)$$

where energy changed $\Delta E_{i} = E_{i + 1} - E_{i}$ and the critical value $S\left( {E_{i} } \right)$ related to the initial energy layer is

$$S\left( {E_{i} } \right) = \left\{ {\begin{array}{*{20}l} {0.2E_{i} - 39.5\;{\text{MeV}}} \hfill & {if\;\;E_{i} < 110\;{\text{MeV}}} \hfill \\ {0.1E_{i} - 28.5\;{\text{MeV}}} \hfill & {if\;\;110 \le E_{i} < 150\;{\text{MeV}}} \hfill \\ {0.05E_{i} - 21\;{\text{MeV}}} \hfill & { if\;\;E_{i} \ge 150\;{\text{MeV}}} \hfill \\ \end{array} } \right.$$

In routine clinical practice, descending energy layer switching interval in an IMPT treatment field is normally less than 10 MeV. As a result, 0.7 s would be sufficient (as an average ELST within 10 MeV) to estimate a relatively accurate delivery time for most clinical scenarios**.**

## B: Spot scanning switching time modeling

The “dead-time” between the spot switching, or magnetic preparation time is about 1 ms which can be directly acquired from the log file from the test field experiment described in 2.C.2. Based on the ten clinical treatment fields and the y-direction experimental test field’s log files, spot switching time (SSWT) can be plotted as a function of spot traveling distance in x-direction and y-direction. Then, these data can be fitted by different curves for SSWT modeling.

**Fig. 5:**
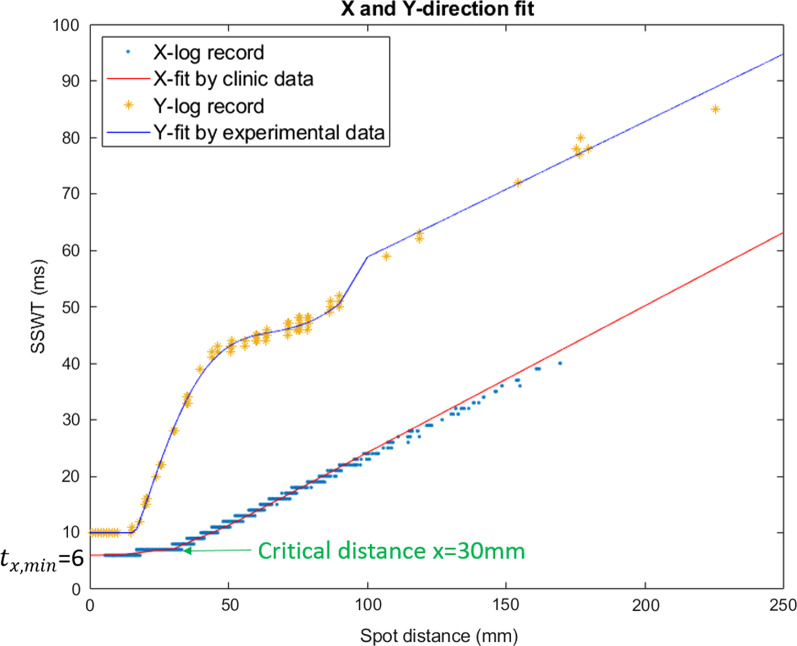
Spot switching time fitting in x and y direction. Spot scanning time modelling in x and y directions: x-direction data fitted by the modified Softplus function from ten clinical cases and y-direction experimental data fitted by a three-piecewise polynomial using test fields. The slope of the fit line is the reciprocal of the spot scanning speed. X-direction speed is faster than y-direction. The x-fit curve has a turning point at x = 30 mm and the baseline is 6 ms

From the comparison in Fig. 5, x-direction speed is faster than y-direction. The IBA scanning control system places the spots consecutively on a line along the fast scanning direction [18]. Therefore, this line is the x-direction.

The spot switching time in x-direction ${t}_{x}$ can be described by a modified softplus function [29] (Fig. 5) with respect to its scan distance *x* mm:

$$t_{x} = \frac{{t_{x,max} }}{{x_{max} - x_{min} }}\ln \left( {1 + e^{x - 30} } \right) + e^{{ - \frac{1}{2}\left( {\frac{x - 30}{{10}}} \right)^{2} }} + t_{x, min} \;\;({\text{ms}})$$

where *x* is scanning distance given in mm and used without a unit, time is given in ms.

This function contains of two parts: smooth part and random part. Smooth part $\ln \left( {1 + e^{x - 30} } \right)$ is a Smooth ReLU function [30] that shifts right to a critical distance 30 mm. The random part is a Gaussian distribution centered at this critical distance 30 mm. $x_{max}$ and $x_{min}$ are maximal and minimal distance (given in mm and used without a unit) at the same line within a treatment field. $t_{x.max}$ and $t_{x,min}$ are maximal and minimal spot switching time (given in ms and used without a unit) at the same line in a treatment field. From the log file (“X-log record” in the Fig. 5), this $t_{x, min} = 6$ (given in ms and used without a unit). And the normalization coefficient $t_{x, max} /(x_{max} - x_{min} ) = 0.26$ (given in s/m and used without a unit) as an average from the 2.C.2’s ten clinical IMPT beams (see Additional file 1: Table s4).

The spot switching time along the y-direction only ($t_{y}$) can be modeled by a piecewise polynomial (Fig. 5):

$$t_{y} = \left\{ {\begin{array}{*{20}l} {10} \hfill & {(y < 15.35 )} \hfill \\ {\mathop \sum \limits_{n = 0}^{7} p_{n} y^{n} } \hfill & {(15.35 \le y < 100 )} \hfill \\ {0.2399y + 34.809} \hfill & {\left( {y \ge 100 } \right)} \hfill \\ \end{array} } \right.\;\;\left( {{\text{ms}}} \right)$$

where y is scanning distance given in mm and used without a unit, and the 7th degree polynomial’s coefficients in 16 digital precision are:

$$p_{0} = 3.701164361860537$$

$$p_{1} = - 1.431281202229966$$

$$p_{2} = 0.1760201780078124$$

$$p_{3} = - 4.965471530006315 \times 10^{ - 3}$$

$$p_{4} = 6.520478442495668 \times 10^{ - 5}$$

$$p_{5} = - 4.408172810657032 \times 10^{ - 7}$$

$$p_{6} = 1.484166290834395 \times 10^{ - 9}$$

$$p_{7} = - 1.967097213355599 \times 10^{ - 12}$$

Spot scanning in the diagonal direction (both x and y direction) mostly happens in line switching, the line switching time ($t_{RSW}$) is determined by either the time in x-direction ($t_{x\_rsw}$) or the time in y-direction ($t_{y\_rsw}$) whichever takes longer (see Additional file 1: Table s5). Thus, the diagonal switching time or line switching time can be written as:

$$t_{RSW} = {\text{max}}\left( {t_{x\_rsw} , t_{y\_rsw} } \right)$$

Therefore, the total SSWT can be written as

$$T_{SSW} = \mathop \sum \limits_{i = 1}^{{N_{layer} }} \mathop \sum \limits_{j = 1}^{{N_{burst}^{i} }} \mathop \sum \limits_{r = 1}^{{N_{line}^{i,j} }} \left( {t_{RSW}^{i,j,r} + \mathop \sum \limits_{k = 1}^{{N_{spot}^{i,j,r} - 1}} t_{x}^{i,j,r,k} } \right)\;\;({\text{ms}})$$

where $N_{line}^{i,j}$ is the total number of lines in *j*th burst of *i*th layer and $N_{spot}^{i,j,r}$ is the total number of spots in *r*th line.

## C: Spot spill time model

Spot spill time model includes two components: maximum charges per pulse and prediction of pulse number per spot in each radiation burst.

## C.3a maximum charges per pulse

**Fig. 6:**
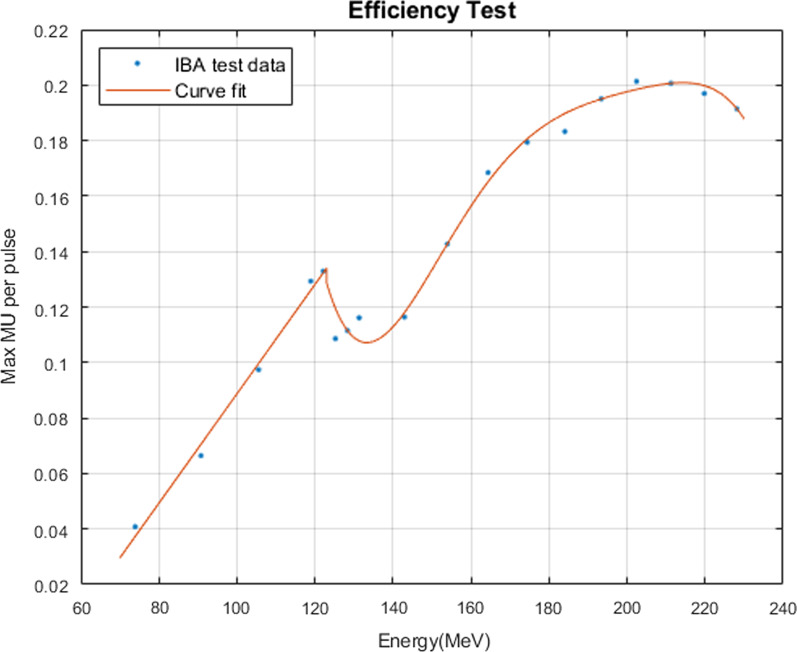
The maximal efficiency as a function of energy layer

The charges per pulse are determined by V_Dee_ setpoint. The maximum charges per pulse at V_Dee_ = 100% were plotted as a function of energy layers (Fig. 6). The curve’s different slope and shape as a function of the energy layers indicate that different degrader material was applied during the energy layer selection. Thus, it resulted in different scattering and beamline transmission efficiency. In this section, a two-piecewise polynomial function can be used to fit the data for maximum efficiency model.

$$v_{i} = \left\{ {\begin{array}{*{20}l} {0.001971\frac{{E_{i} }}{{1\;{\text{MeV}}}} - 0.1084} \hfill & {(E_{i} < 123\;{\text{MeV}})} \hfill \\ {\mathop \sum \limits_{n = 0}^{5} a_{n} \left( {\frac{{E_{i} }}{{1\;{\text{MeV}}}}} \right)^{n} } \hfill & {\left( {E_{i} \ge 123\;{\text{MeV}}} \right)} \hfill \\ \end{array} } \right.\;\;\left( {\text{MU/pulse}} \right)$$

where ${E}_{i}$ is the energy (MeV) in layer i and the 6th degree polynomial’s coefficients in 16 digital precision are:

$$a_{0} = 34.91785611467123$$

$$a_{1} = - 0.9845329482724059$$

$$a_{2} = 0.01098029030171986$$

$$a_{3} = - 0.00006048892122110666$$

$$a_{4} = 0.000000165088904656624$$

$$a_{5} = - 0.0000000001789254400704347$$

## C. 3b Prediction of pulse number per spot in each radiation burst

The average MU percentage weighting per spot is about 60.9% in the first radiation burst, 33.0% in the second burst, 6.1% in the 3rd burst (Table 1).

**Table 1:** Burst percentage result from 64 energy layers of the experiment designed in 2.C.3

	Burst 1 (%)	Burst 2 (%)	Burst 3 (%)
Average MU weighting	60.9	33.0	6.1
Standard deviation	2.6	5.0	4.0

Then, for a certain spot, the number of pulses to be delivered in a radiation burst can be calculated through dividing the MU of the burst by the maximum charges per pulse (maximum efficiency, 3C.3.a). In other words, the pulse number to be delivered for spot *k*, burst *j* and energy layer *i*, can be written as:

$$n_{pulse}^{i,j,k} = \frac{{MU_{plan}^{i,j,k} S_{j} }}{{v_{i} }} \left( {j = 1,2,3} \right)$$

where $MU_{plan}^{i,j,k}$ is the plan MU to be delivered for spot *k* in layer *i* and burst *j*, $S_{1} = 60.9\%$, $S_{2} = 33.0\%$ and $S_{3} = 6.1\%$ are the percentage of the MU delivered in burst *j* = 1,2,3 respectively (average value from Table 1), $v_{i}$ is the maximal efficiency defined in 2.C.3a, and $x$ is the ceiling function that maps the input number to the least integer greater than or equal to *x*. In clinic, $MU_{plan}^{i,3,k} S_{3}$ is very small that is less than $v_{i}$. All spots are delivered as a single pulse in 3rd burst based on this model. For j = 1 and 2, this $n_{pulse}^{i,j,k}$ in general, could be between 2 and 6.

## D: Burst switching modeling

**Fig. 7:**
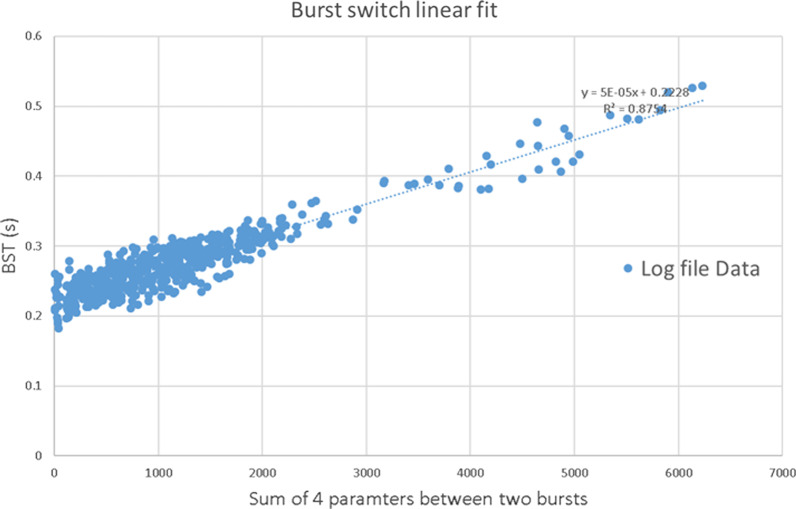
The burst switching time fitting result. Linear regression for BST through ten clinical IMPT beam cases and R^2^ = 0.8754

The machine log file size is proportional to the irradiation number of the radiation burst ($N_{SSW}^{i,j} + N_{pulse}^{i,j}$) (see Additional file 1: Fig. s9) and the command file size is proportional to the number of the pulses and spots ( $N_{pulse}^{i,j + 1} + N_{spot}^{i,j + 1}$) to be delivered in the following burst (see Additional file 1: Fig. s10). The data transmission speed may vary depending on the workstation’s inbound and outbound traffic and device settings and configurations [31]. Therefore, we use a linear regression representing the BST (extracted from the analysis of the log files) depending on the sum of 4 parameters $(N_{SSW}^{i,j} + N_{pulse}^{i,j} + N_{pulse}^{i,j + 1} + N_{spot}^{i,j + 1} )$ in the next two bursts (Fig. 7).

The BST between burst *j* and *j* + *1* in the layer *i* is given by:

$$t_{bsw}^{i,j} = \mu (N_{SSW}^{i,j} + N_{pulse}^{i,j} + N_{pulse}^{i,j + 1} + N_{spot}^{i,j + 1} ) + 0.2228\;{\text{s}}$$

where $\mu$ is the data transmission speed coefficient between two bursts (slope of the linear fit in the Fig. 7), which is a constant $5 \times 10^{ - 5}$ s, $N_{SSW}^{i,j}$ is the total spot switching number in the previous burst which is defined in 2.C.4, $N_{pulse}^{i,j}$ is the total number of pulses in the previous burst. And $N_{pulse}^{i,j + 1}$ is the total number of pulses in next burst. $N_{spot}^{i,j + 1}$ is the total number of spots in the next burst, 0.2228 s is the burst switching “dead time” (vertical intercept of the linear fit in the Fig. 7). $N_{pulse}^{i,j}$ and $N_{pulse}^{i,j + 1}$ can be obtained by the spot number prediction model in 3.C.3b.

## E: Validation test using clinical treatment fields

**Fig. 8:**
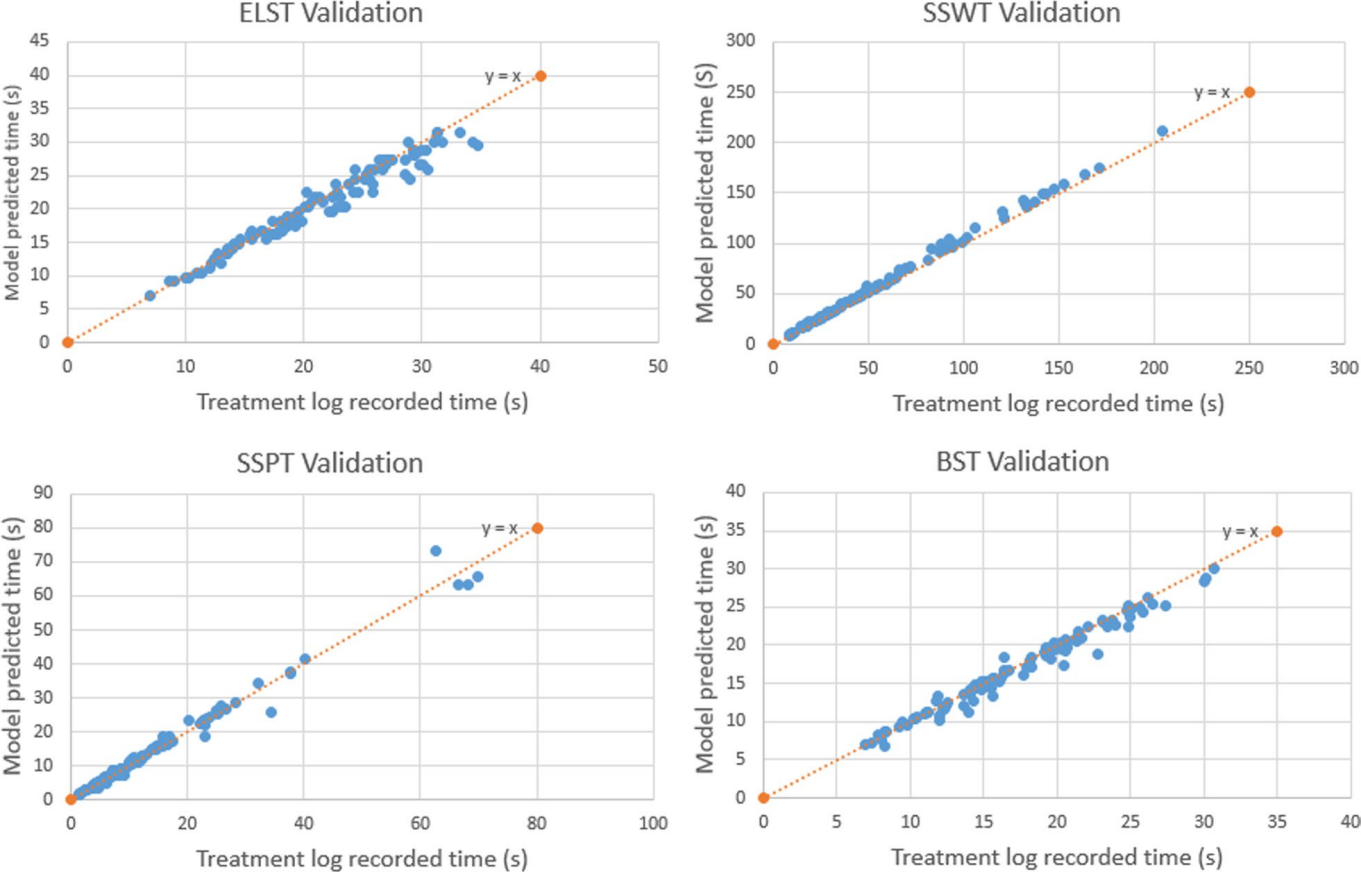
Validation result of beam delivery time for each component. Comparison of $T_{LSW}$, $T_{SSW}$, $T_{SSP}$ and $T_{BSW}$ between the model prediction and the actual treatment time from machine log files for 102 individual treatment fields

This BDT prediction model was validated through 102 IMPT clinical treatment fields. Figure 8 shows the comparison of the predicted delivery time for $T_{LSW}$, $T_{SSW}$, $T_{SSP}$, $T_{BSW}$ and actual recorded delivery time. The average difference of each time component from the machine log files is listed in Table 2.

**Table 2:** Average difference of each time component compared with log files

Time component	Relative difference in percentage	Absolute difference (s)
ELST	(− 3.1 ± 5.7)% ([− 8.8%, 2.6%])	− 0.8 ± 1.4
SSWT	(5.9 ± 3.9)% ([2.0%, 9.8%])	3.1 ± 2.9
SSPT	(2.6 ± 8.7)% ([− 6.1%, 11.3%])	0.2 ± 1.8
BST	(− 2.3 ± 5.3)% ([− 7.6%, 3.0%])	− 0.5 ± 0.9
Total BDT	(2.1% ± 3.0)% ([− 0.9%, 5.1%])	2.1 ± 3.6

**Fig. 9:**
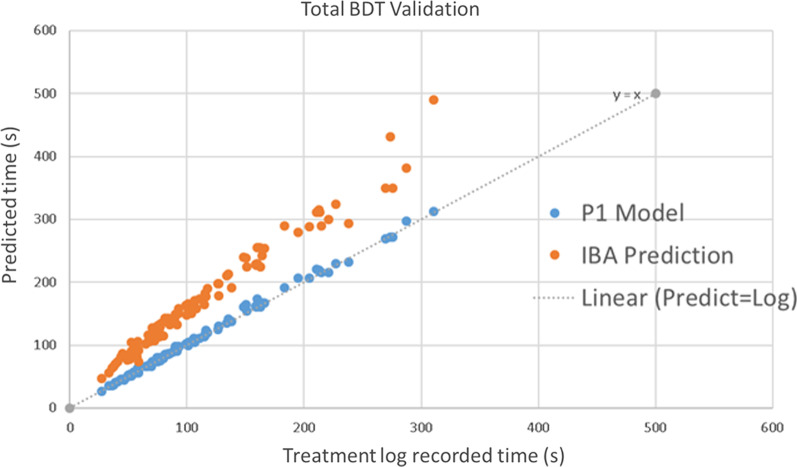
Validation result of total beam delivery time compared with IBA. Comparison of $T_{BDT}$ between the model prediction and the actual treatment time from machine log files for 102 individual treatment fields

The average total BDT was within (2.1 ± 3.0)% ([− 0.9%, 5.1%]) difference compared to the machine log files (Fig. 9), which is a significant improvement from the original BDT calculation from IBA ProteusONE®: (58 ± 15)% ([43%, 73%]). The standard deviation of the absolute time is 3.6 s for the in-house model and 26 s for IBA commercial system’s prediction. The standard deviation of predicted BDT was reduced by 22.6 s using this in-house model compared with IBA system’s prediction (see Additional file 1: Table s6).

Also, we obtain the average percentage of time spent on layer switching, spot switching, spot spill, and burst switching from 102 clinical cases (Table 3). Spot switching is the most time-consuming.

**Table 3:** The average percentage of the beam delivery time spent in layer switching, spot switching, spot spill and burst switching from 102 clinical cases

Delivery type	The average percentage of the total predicted time from the in-house model (%)	The average percentage of the total recorded time from the log file (%)
Layer switching	22.12	23.19
Spot switching	49.31	47.33
Spot spill	11.35	11.31
Burst switching	17.20	18.17

## F: Interplay effect evaluation

**Fig. 10:**
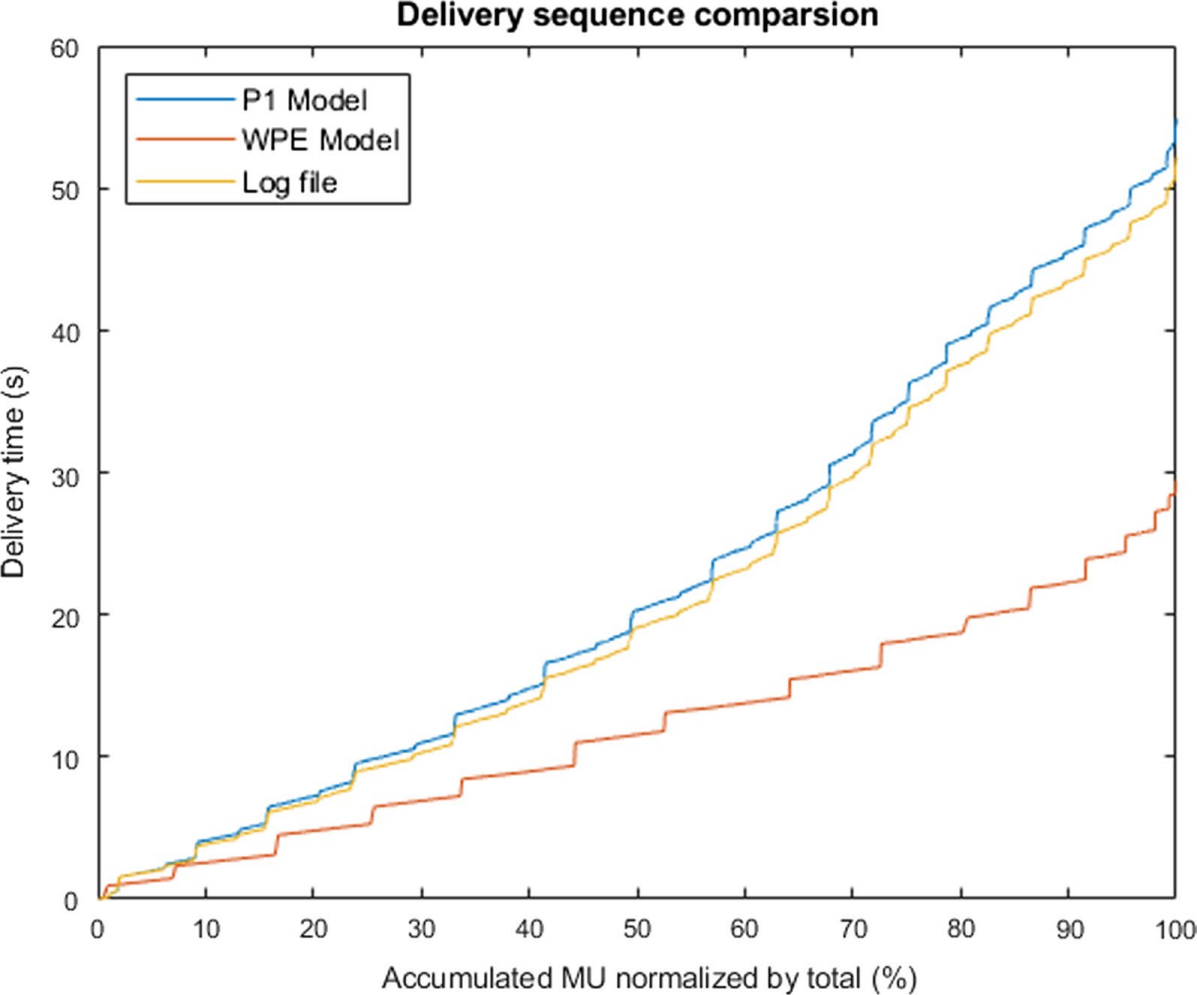
Delivery sequence comparison for a lung SFUD field. The in-house ProteusONE® model (labeled by P1 in the figure) was compared with log files record and a simplified standard delivery sequence model from West German Proton Therapy Center in Essen (WPE)

**Fig. 11:**
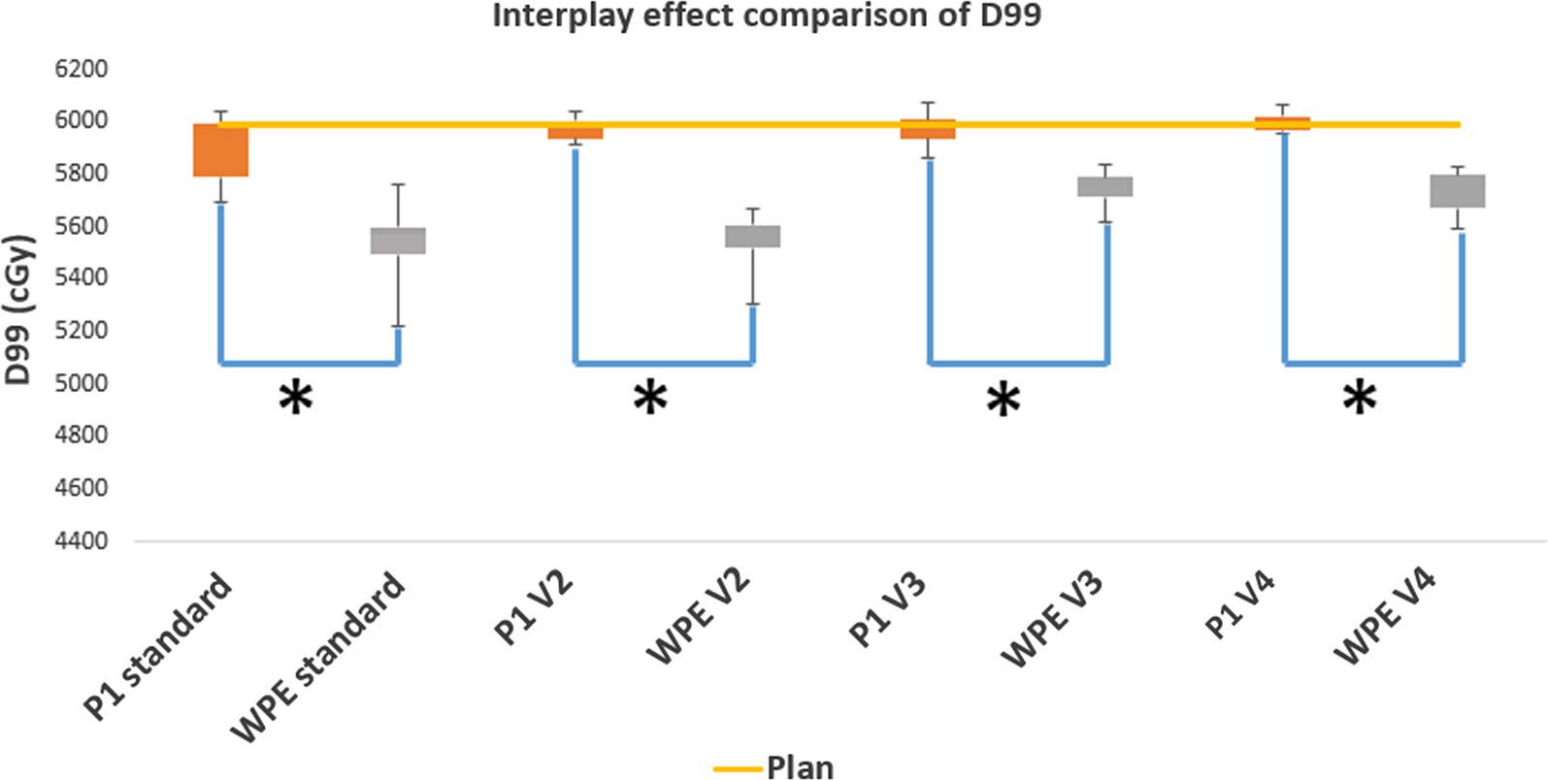
D99 boxplot comparison based on the interplay effect evaluation. Compared to the non-burst mechanism, a conventional cyclotron system, WPE model, the in-house ProteusONE® model (labeled as P1) simulation result showed better mitigation in the interplay effect compared to the WPE model simulation for standard and volume repainting technique (V2: Two volumetric repainting; V3: Three volumetric repainting; V4: Four volumetric repainting). * indicates a significant difference in comparison with variable values at *p* < 10^−5^

The delivery sequence comparison among this machine-specific model (P1 model), log files record and standard delivery WPE model for an example treatment field (see 2.E) was demonstrated in Fig. 10. The machine-specific delivery sequence showed a better agreement with logfile compared to the WPE model simulation. Compared to the reconstructed 4D dynamic dose from the standard and volumetric repainting (N = 2, 3, 4) delivery, the in-house machine-specific ProteusONE® model reconstructed dose showed a better interplay effect mitigation compared to the WPE model because of the burst mechanism (Fig. 11 and Additional file 1: Fig. s11). The D99 of the initial plan was 5990 cGy. The mean and standard deviation of D99 calculated from the standard delivery interplay effect was 5894 ± 125 cGy based on the in-house machine-specific model, and 5539 ± 159 cGy based on the WPE model, respectively. In each comparison groups of different volumetric repainting, the machine-specific ProteusONE® model also showed a better interplay effect mitigation (*p* value < 0.001).

## Discussion

This is the very first study to develop an accurate beam delivery sequence and time prediction model for the proton therapy system based on the IBA ProteusONE®’s superconducting synchrocyclotron accelerator. It presents a detailed methodology to isolate and determine the beam delivery parameter experimentally, which could be used to model other proton systems, including cyclotron, synchrotron, or other synchrocyclotron accelerators (the synchrocyclotron systems from other vendors may have a different delivery sequence that needs to be further investigated). This machine-specific model divides the total BDT into four main components (energy layer switching time, burst switching time, spot scanning switching time, and spot spill time) where BST, which is specific to the IBA S2C2, has not been studied before. The dynamic of each scanning magnet depends on its physical properties (e.g. location in the beamline, proximity to iron pieces, etc.) and the calibration (that includes safety margins, especially for large displacements in Y). In the IBA ProteusONE® system, the magnets are placed sequentially. It is therefore expected that their properties are different. For instance, the amplitude of the deflection is not the same in both magnets for the same displacement at the isocenter.

Our community always has an impression that the largest contribution to the total BDT is the ELST [32]. For example, about decades ago, the IMPT treatment delivery efficiency bottleneck was the ELST (~ 2 s to 10 s) [32]. However, this is not the case anymore for IBA’s synchro-cyclotron system. In this new generation of the proton therapy system, the ELST only occupied 23% of BDT (Table 3). In order to further improve the treatment delivery efficiency of IMPT, several approaches were proposed, such as the energy reduction algorithm [32], ridge filter [33], and faster energy layer selection system [33]. Based on this in-house model of IBA ProteusONE®, the spot switching time is now occupying the majority portion of the total BDT 47% (Table 3). This is because the new mechanism of iterative layer delivery via three radiation bursts effectively increased the number of spots to be delivered by three times. Now SSWT becomes a new bottleneck. These new findings will lead to the next generation of development, such as the fast spot scanning system and the spot reduction optimization algorithm for IMPT [34]. The performances reported in this section may be valid for the current version installed at this moment. The new development in the spot scanning hardware could significantly reduce the SSWT in the more recent IBA ProteusONE® version.

The interplay effect evaluation result indicates that this in-house machine-specific model can predict not only the total treatment time but also the spot delivery sequence because each beam parameter was precisely modeled. Even though some proton systems are from the same vendor, such as IBA, their delivery sequence might be totally different. For example, IBA products ProteusONE® and ProteusPLUS® have a different delivery mechanism due to the accelerator design, which resulted in a different delivery sequence and time (Fig. 10) and a different interplay effects estimation (Fig. 11 and Additional file 1: Fig. s11). As we discussed in 2.A and 2.E, ProteusONE® system internal burst mechanism plays a role like an adaptive layer repainting technique. The time-scale of the burst mechanism is very similar to the layer repainting’s time-scale. Thus, it has a positive impact on the interplay effect mitigation (Fig. 11 and Additional file 1: Fig. s11). The result shows this in-house model can be used to precisely describe the iterative layer delivery algorithm which was introduced in the IBA ProtuesONE®. ProteusONE® effectively has more repainting of the spot due to its internal burst mechanism. Such iterative layer delivery mechanism could reduce the interplay effect as a dedicated rescanning technique. Thus, based on our machine-specific model, we predicted less volumetric repainting interplay effect with ProteusONE® than the prediction made by WPE model which is based on ProteusPLUS® system delivery sequence model, suggesting the importance of using the correct machine model. As a result, each facility should have the machine-specific beam delivery model.

Besides, an accurate beam delivery sequence and BDT prediction model would guide the next treatment technique revolution [11]. For example, based on this model, the community is able to access the delivery time component that was not reported in the previous publication such as ascending energy layer switching. This extra information could play an important role in the development of the spot-scanning proton arc (SPArc) therapy [35], where the SPArc plan has hundreds of energy layers, including numerous ascending switches. A precise BDT and deliver sequence model developed in this study would be able to estimate a more realistic proton arc delivery time and provide guidance in the technology development in this direction, such as spot number and energy layer number reduction and redistribution [35], energy layer sequencing optimization algorithm [36] as well as the proton arc gantry rotation controllers. If IBA is able to optimize the ascending energy direction, SPArc treatment delivery time could be further reduced.

The absolute and relative difference between the BDT prediction from the in-house model and IBA’s treatment console indicated the potential improvements could be made when the therapists work on the patient's treatment schedule.

To test if this in-house IBA ProteusONE® delivery time and sequence model could help better estimate the treatment irradiation time and improve the daily patient treatment throughput, a total of 12 cases from the four disease sites such as prostate, head and neck, lung, and chest wall cancer were retrospectively selected. The result (See Additional file 1: Fig. s12) shows that among the common patient population, the head and neck, lung, and chest wall cancer case might benefit more from this in-house model during the patient’s scheduling which could save about 3 min per fraction. More specifically, the differences are 176 s (56%), 177 s (55%), and 157 s (32%) for head and neck, lung, and chest wall cancer, respectively. In comparison, the difference in prostate cancer cases is only 59 s (48%). Although 3 min per patient may not be a significant impact to the clinical operation, total of 20 patient per room per day may result in an hour difference or 2 patient treatment slots.

One limitation of this model is that it is not able to take system’s daily variation into account during the calculation or simulation. The daily variation happens due to the synchrocyclotron status and ion source efficiency, which could slightly impact the statistical uncertainties and treatment delivery time. To investigate the impacts from the daily variations, we track a completed treatment of a head and neck case with 25 fractions across 38 days (See the Additional file 1: Fig. s13 and s14). The standard deviation of each time component are small throughout the treatment course: ELST (0.41 s (1.19%)), BST (0.51 s (1.64%)), SSWT (1.44 s (0.70%)), SSPT (0.65 s (1.61%)) and total BDT (1.52 s (0.49%)). This example shows the influence of the daily variation on the actual treatment delivery time is very small. Although this in-house ProteusONE® BDT model cannot consider such daily variations of synchrocyclotron status since this information is not accessible to clinical users, the overall daily variation could be negligible. The measurements were based on many tests and experiments in different days that smoothed out the daily variations during the modeling. Overall, this new BDT model’s prediction time could reach (2.1 ± 3.0)% for the IBA ProteusONE® system where such accuracy should be able to meet the needs of most clinical applications. Other institutions can adopt the method introduced in this paper to determine their individual accelerator parameters. Even a different IBA ProteusONE® facility needs to re-estimate or at least independently validate the machine-specific model because the software and hardware may not be identical. Most importantly, this work recommends using a machine-specific model for each institution because each machine might have its own specific delivery sequence due to the different hardware or software versions.

## Conclusion

An experimental modeling approach was developed to determine relevant proton therapy operational parameters for the new synchrocyclotron accelerator-based proton beam therapy system, IBA ProteusONE®. This method could be applied to other proton institutions for BDT modeling. For the first time, a new parameter burst switching time (BST) was taken into account in the model for accurate beam delivery time prediction. This validated model could be potentially used in the clinic to evaluate the motion interplay effect as well as the daily patient treatment throughput. Moreover, the results from the study could be potentially used as a reference to guide the optimal design of the proton beam scanning controller and delivery sequence for this system.

## Supplementary Information

**Additional file 1.** An intuitive example of the spot delivery sequence based on the iterative layer delivery algorithm. **Fig. s1.** The control interface of the IBA ProteusONE in Beaumont Proton Therapy Center to predict the BDT. **Fig. s2.** A morning machine look-up table reflects the relationship between uncertainty and VDee. **Table s1.** Energy layer switching experiment design. **Fig. s3.** The relationship of ion numbers per MU. **Table s2.** Spot scanning diagonal speed experiment design. **Fig. s4.** A morning machine look-up table reflects the relationship between mean charge per pulse and VDee. **Table s3.** Burst percentage and efficiency test for different gantry angle. **Fig. s5.** An example of the log file package from the IBA ProteusONE. **Fig. s6.** The proton system records the radiation delivery information in the csv file. **Fig. s7.** The command file size reflects the number of pulse and spot to be delivered in next burst. **Fig. s8.** Two-field SFUD plans were generated in TPS. **Table s4.** The normalization coefficient of ten treatment fields. **Table s5.** The diagonal switching time experiment result. **Fig. s9.** The record log file size. **Fig. s10.** The command file size. **Table s6.** Deviation comparison with log files. **Fig. s11.** DVH band curve of interplay effect. **Fig. s12.** The model prediction time compared to the actual irradiation time. **Fig. s13.** Time component changes. **Fig. s14** Total BDT changes.

## Data Availability

The datasets used and/or analyzed during the current study are available from the corresponding author on reasonable request.

## Abbreviations

BDT: Beam delivery time sequence
BST: Burst switching time sequence
ELST: Energy layer switching time sequence
SSPT: Spot spill time sequence
SSWT: Spot switching time sequence
PBS: Pencil beam scanning
IMPT: Intensity modulated proton therapy
SPArc: Spot-scanning proton arc
MU: Monitor unit
IBA: Ion Beam Application Company

## List of symbols

$T_{BDT}$: Total beam delivery time
$T_{BSW}$: Total burst switching time
$T_{LSW}$: Total energy layer switching time
$T_{SSW}$: Total spot switching time
$T_{SSP}$: Total spot spill time
$N_{vol}$: Total number of volumetric repainting in the beam
$N_{layer}$: Total number of layers in the beam
i: Energy layer index
j: Burst index
r: Line index
K: Spot index
$N_{burst}^{i}$: Total number of bursts in the layer *i*
$N_{row}^{i,j}$: Total number of rows in the layer *i*, burst *j*
$N_{spot}^{i,j}$: Total number of spots in the layer *i*, burst *j*
$N_{pulse}^{i,j}$: Total number of pulses in the layer *i*, burst *j*
$T_{SSW}^{i,j}$: Total spot switch time in the layer *i*, burst *j*
$$N_{SSW}^{i,j}$$: $$N_{SSW}^{i,j} = T_{SSW}^{i,j} /1ms$$
$N_{spot}^{i,j,r}$: The total number of spots in in the layer *i*, burst *j*, line *r*
$n_{pulse}^{i,j,k}$: Total number of pulses in the layer *i*, burst *j* and spot *k*
$E_{i}$: Energy (MeV) in layer *i*
$\Delta E_{i}$: Energy difference between layer *i* and layer *i* + *1*
$S\left( {E_{i} } \right)$: Critical value related to the layer *i*
V_Dee_: The voltage between the Dees
$v_{i}$: Maximal efficiency at layer *i*
$MU_{plan}^{i,j,k}$: Plan MU in the layer *i*, burst* j* and spot *k*
$S_{j}$: Percentage of the MU to be delivered in burst *j*
$a$: Ceiling function maps the input number to the least integer greater than or equal to *a*.
$t_{lsw}^{i}$: Switch time from layer *i* to layer *i* + *1*
${\varvec{\mu}}$: Transmission data efficiency between two bursts
x: Scan distance in x-direction
$x_{min}$: Minimal distance at the same line
$x_{max}$: Maximal distance at the same line
$t_{x}^{i,j,r,k}$: Switch time of spot k to spot *k* + 1 in the layer *i*, burst *j*, line *r*
$t_{y}$: Spot switch time at the y-direction of the scan distance y
$$t_{baseline}$$: Baseline of $$t_{y}$$
$t_{x,min}$: Minimal scan switch time at the same line
$t_{x.max}$: Maximal scan switch time at the same line
$t_{x\_rsw}$: Assumed spot switch time in x direction when line switches
$t_{y\_rsw}$: Assumed spot switch time in y direction when line switches
$t_{RSW}^{i,j,r}$: Switching time of line r to line *r* + 1 in the layer *i*, burst *j*

## References

[1] Soukup M, Fippel M, Alber M (2005). A pencil beam algorithm for intensity modulated proton therapy derived from Monte Carlo simulations. Phys Med Biol.

[2] Zheng Y, Newhauser W, Fontenot J, Taddei P, Mohan R (2007). Monte Carlo study of neutron dose equivalent during passive scattering proton therapy. Phys Med Biol.

[3] Zhang X, Li Y, Pan X (2010). Intensity-modulated proton therapy reduces the dose to normal tissue compared with intensity-modulated radiation therapy or passive scattering proton therapy and enables individualized radical radiotherapy for extensive stage IIIB non-small-cell lung cancer: a virtual clinical study. Int J Radiat Oncol.

[4] Li X, Kabolizadeh P, Yan D (2018). Improve dosimetric outcome in stage III non-small-cell lung cancer treatment using spot-scanning proton arc (SPArc) therapy. Radiat Oncol.

[5] Liu C, Bhangoo RS, Sio TT (2019). Dosimetric comparison of distal esophageal carcinoma plans for patients treated with small-spot intensity-modulated proton versus volumetric-modulated arc therapies. J Appl Clin Med Phys.

[6] Li H, Li Y, Zhang X (2012). Dynamically accumulated dose and 4D accumulated dose for moving tumors: dynamic dose and 4D dose. Med Phys.

[7] Li H, Zhang X, Li Y, Zhu RX (2019). An analytical model for the upper bound estimation of respiratory motion-induced dose uncertainty in spot-scanning proton beam therapy. Med Phys.

[8] Liu H, Chang JY (2011). Proton therapy in clinical practice. Chin J Cancer.

[9] Goitein M, Jermann M (2003). The relative costs of proton and X-ray radiation therapy. Clin Oncol.

[10] Fornell D. Trends in proton therapy-faster therapy delivery, single room installs. Imaging Technology News. November 1, 2018.

[11] Farr JB, Flanz JB, Gerbershagen A, Moyers MF (2018). New horizons in particle therapy systems. Med Phys.

[12] Manuel B, Schillo M, Schultheiss J, Cruz L. Compact proton therapy system with energy selection onboard a rotatable gantry. Published online March 15, 2016. https://patents.google.com/patent/US9283407B2/en.

[13] Henrotin S, Abs M, Forton E, et al. Commissioning and testing of the first IBA S2C2. In: Proceedings of 21st international conference on cyclotrons and their applications. 2016.

[14] Pidikiti R, Patel BC, Maynard MR (2018). Commissioning of the world’s first compact pencil-beam scanning proton therapy system. J Appl Clin Med Phys.

[15] IBA Reports Full Year 2019 Results; 2020. https://iba-worldwide.com/content/iba-reports-full-year-2019-results.

[16] Silva LO. ProteusONE training-part 1: system description. Presented at: ASTRO 2017; 2017.

[17] Shen J, Tryggestad E, Younkin JE (2017). Technical note: using experimentally determined proton spot scanning timing parameters to accurately model beam delivery time. Med Phys.

[18] Pfeiler T, Bäumer C, Engwall E, Geismar D, Spaan B, Timmermann B (2018). Experimental validation of a 4D dose calculation routine for pencil beam scanning proton therapy. Z Für Med Phys.

[19] Kleeven W, Abs M, Forton E, et al. The IBA superconducting synchrocyclotron project S2C2. In: Proceedings of Cyclotrons 2013; 2013:115–119. https://accelconf.web.cern.ch/Cyclotrons2013/papers/mo4pb02.pdf.

[20] Zenklusen SM, Pedroni E, Meer D (2010). A study on repainting strategies for treating moderately moving targets with proton pencil beam scanning at the new Gantry 2 at PSI. Phys Med Biol.

[21] Poulsen PR, Eley J, Langner U, Simone CB, Langen K (2018). Efficient interplay effect mitigation for proton pencil beam scanning by spot-adapted layered repainting evenly spread out over the full breathing cycle. Int J Radiat Oncol.

[22] Paganetti H, Bortfeld T. Proton therapy. In: New technologies in radiation oncology. Springer; 2006. p. 345–363.

[23] Hodgdon ML (1988). Mathematical theory and calculations of magnetic hysteresis curves. IEEE Trans Magn.

[24] Liu G, Hu F, Ding X (2019). Simulation of dosimetry impact of 4DCT uncertainty in 4D dose calculation for lung SBRT. Radiat Oncol.

[25] Lomax A. SFUD, IMPT, and plan robustness. In: Particle radiotherapy. Springer; 2016. p. 169–194.

[26] Magro G, Mein S, Kopp B (2021). FRoG dose computation meets Monte Carlo accuracy for proton therapy dose calculation in lung. Phys Med.

[27] OpenREGGUI. Ion beam application https://openreggui.org/.

[28] Levin WP, DeLaney TF. Charged particle radiotherapy. In: Clinical radiation oncology. 4th ed. Elsevier; 2015. p. 358. https://www.elsevier.com/books/clinical-radiation-oncology/9780323240987.

[29] Zheng H, Yang Z, Liu W, Liang J, Li Y. Improving deep neural networks using softplus units. In: 2015 International joint conference on neural networks (IJCNN); 2015. p. 1–4. 10.1109/IJCNN.2015.7280459.

[30] Smooth Rectifier Linear Unit (SmoothReLU) Forward Layer. In: Developer guide for intel data analytics acceleration library; 2017.

[31] Bezaire DL, Owens SJ, Hronek DJ. System for transmitting messages, between an installed network and wireless device. Published online 1998. https://patents.google.com/patent/US5758088A/en.

[32] Cao W, Lim G, Liao L (2014). Proton energy optimization and reduction for intensity-modulated proton therapy. Phys Med Biol.

[33] Courneyea L, Beltran C, Tseung HSWC, Yu J, Herman MG (2014). Optimizing mini-ridge filter thickness to reduce proton treatment times in a spot-scanning synchrotron system. Med Phys.

[34] Water van de S, Belosi MF, Albertini F, Winterhalter C, Weber DC, Lomax AJ (2020). Shortening delivery times for intensity-modulated proton therapy by reducing the number of proton spots: an experimental verification. Phys Med Biol.

[35] Ding X, Li X, Zhang JM, Peyman K, Stevens C, Yan D (2016). Spot-scanning proton arc (SPArc) therapy: the first robust and delivery-efficient spot-scanning proton arc therapy. Int J Radiat Oncol Biol Phys.

[36] Liu G, Li X, Zhao L (2020). A novel energy sequence optimization algorithm for efficient spot-scanning proton arc (SPArc) treatment delivery. Acta Oncol.

