# Supplementary material for: Developing an accurate model of spot-scanning treatment delivery time and sequence for a compact superconducting synchrocyclotron proton therapy system
Source: Radiat Oncol. 2022 May 7;17:87. doi: 10.1186/s13014-022-02055-w (PMC9077949; doi:10.1186/s13014-022-02055-w)
Supplement: Supplementary file 1 — Additional file 1. An intuitive example of the spot delivery sequence based on the iterative layer delivery algorithm. Fig. s1. The control interface of the IBA ProteusONE in Beaumont Proton Therapy Center to predict the BDT. Fig. s2. A morning machine look-up table reflects the relationship between uncertainty and VDee. Table s1. Energy layer switching experiment design. Fig. s3. The relationship of ion numbers per MU. Table s2. Spot scanning diagonal speed experiment design. Fig. s4. A morning machine look-up table reflects the relationship between mean charge per pulse and VDee. Table s3. Burst percentage and efficiency test for different gantry angle. Fig. s5. An example of the log file package from the IBA ProteusONE. Fig. s6. The proton system records the radiation delivery information in the csv file. Fig. s7. The command file size reflects the number of pulse and spot to be delivered in next burst. Fig. s8. Two-field SFUD plans were generated in TPS. Table s4. The normalization coefficient of ten treatment fields. Table s5. The diagonal switching time experiment result. Fig. s9. The record log file size. Fig. s10. The command file size. Table s6. Deviation comparison with log files. Fig. s11. DVH band curve of interplay effect. Fig. s12. The model prediction time compared to the actual irradiation time. Fig. s13. Time component changes. Fig. s14 Total BDT changes. [file 13014_2022_2055_MOESM1_ESM.docx]

**Additional File.**

An intuitive example of the spot delivery sequence based on the iterative layer delivery algorithm.

Based on the treatment plan, a spot has a total 3.80 pC to be delivered by IBA ProteusONE®. The tolerance is within 5% dose accuracy, which is 3.80±0.19pC, for example. A traditional cyclotron’s continuous irradiation will deliver the 3.8pC of that spot within the clinical tolerance before moving to the next spot since it is equipped with the feedback loop to turn off the continuous radiation. However, due to this compact superconducting synchrocyclotron design, the beam is delivered in very short pulses. As opposed to the continuous beam current extracted from an isochronous cyclotron, the time to deliver a pulse is too short for an effective feedback loop during the pulse delivery itself. Delivering a low-dose spot with a single pulse can therefore be subject to unacceptable uncertainty (10% is only given as an example in our paper). When forcing the spots to be delivered in multiple pulses, the feedback loop can be performed on the subsequent pulses. In other words, it would be very unsafe to set the 3.8pC as the target directly as 10 % of overshooting will result in 4.18pC, which is unacceptable. As a result, the iterative layer delivery algorithm is applied to divide the radiation into three bursts. 1^st^ burst was set at 2pC (Figure 1c, left), but it delivered 2.2pC in the 1^st^ burst due to the statistical variations (Figure 1c, middle left). Once the proton system received the delivered dose information from the 1^st^ burst and calculated the remaining charges. In this case, 1.6pC remains to be delivered after the 1^st^ burst (3.8pC - 2.2pC), the algorithm starts calculating the 2^nd^ burst, which was set at 1.5pC (Figure 1c, middle left). However, due to the statistical uncertainties again, it only delivered 1.3pC which was 0.2pC short of the 1.5pC setpoint. As a result, there were 0.3pC remains to be delivered in this spot (total 3.8pC) (Figure 1c, middle right). The algorithm decided to use 3^rd^ burst to finish the irradiation, so it chose 0.2pC as the setpoint. Again in the 3^rd^ burst, it delivered 0.4pC (100% error! from the setpoint 0.2pC). Because the weighting of 3^rd^ burst setpoint (0.2pC) is so small compared to the 3.8pC, the accumulation of the three radiation bursts was 3.9pC which was still within the clinical tolerance of 5% (1^st^ burst delivered 2.2pC, 2^nd^ burst delivered 1.3pC and 3^rd^ burst delivery 0.4pC, total 3.9pC).

In conclusion, this iterative layer delivery algorithm aims to ensure the 3^rd^ burst only contains a relatively small remaining absolute charge as the setpoint, so the relative large uncertainties from the 3^rd^ radiation burst will not exceed the clinical tolerance (5% in figure 3c as an example). As this phenomenon has never been described before, we feel it is crucial for the community to understand this synchrocyclotron accelerator's new mechanism through this study.

To model the layer switch time, we designed the experiment in the following table:

Table s1: Energy layer switching experiment. We choose the small energy interval for -20< ΔE<0 of descending because there is a jump between the step around that range.

| The initial energy (MeV) | The gap of Energy switching (MeV) | End energy (MeV) |
| --- | --- | --- |
| E_0_=100,110,….., 210,220 | ΔE=-1,-2,-3,…..,-19,-20 | E_0_+ ΔE |
| E_0_=70, 80,…,200, 210 | 1 | E_0_+1 |
|  | 2 | E_0_+2 |
|  | 4 | E_0_+4 |
|  | 6 | E_0_+6 |
|  | 8 | E_0_+8 |
|  | 10 | E_0_+10 |
|  | 15 | E_0_+15 |
| E_0_=70, 80,…,180, 190 | 20 | E_0_+20 |
|  | 30 | E_0_+30 |
| E_0_=70, 80,…,170,180 | 40 | E_0_+40 |
| 220 | ΔE=0.5n (n=1,2,…6),  4, 5, 6, 7 | 220+ΔE |

To model the spot line switching time, we design the experiment in the following table:

Table s2: Spot scanning diagonal speed experiment.

| $\boldsymbol{x}_{\boldsymbol{i,j,k+1}}\boldsymbol{-}\boldsymbol{x}_{\boldsymbol{i,j,k}}$ (mm) | $\boldsymbol{y}_{\boldsymbol{i,j,k+1}}\boldsymbol{-}\boldsymbol{y}_{\boldsymbol{i,j,k}}$ (mm) |
| --- | --- |
| +200 | 25 |
|  | 100 |
| -130 | 20 |
| +100 |  |
|  | 25 |
|  | 50 |
|  | 100 |
|  | 200 |
| -90 | 20 |
| +80 |  |
| -50 | 25 |
|  | 50 |
|  | 100 |
| +25 | 25 |
|  | 50 |
| 50 |  |
| 120 | 20 |
| 150 | 25 |
|  | 30 |

To model the spot spill time, we design the experiment in the following table:

Table s3: Burst percentage and efficiency test for different gantry angle. 4 test fields with total 9145 spots for each gantry angle is created below:

| Gantry angle (degree) | Energy layer (MeV) | Spot MU | Spot numbers of each MU value | Spot spacing (cm) |
| --- | --- | --- | --- | --- |
| 45n (n=0,1,…4) | 70+10i (i=0,1,..,15)  and 227 | 0.015 and 0.02 | 62 | 0.4 |
|  |  | 0.06+0.01j (j=0,1,…,34) | 62 |  |
|  |  | 0.41 | 31 |  |
|  |  | 0.43, 0.46 and0.49 | 62 |  |
|  |  | 0.5+0.05j (j=0,1,2,…,100) | 62 |  |
|  |  | 1,2,3,5 and 7 | 62 |  |
|  |  | 10 and 15 | 31 |  |

To obtain the normalization coefficient of the x-direction spot switching time modeling smooth part, we extracted the maximal and minimal distance ($x_{max}$ and $x_{min}$ are given in mm and used without a unit) as well as maxima spot switching time at the same line within a treatment field for ten clinical IMPT beams:

Table s4: The normalization coefficient of ten treatment fields

| Treatment field No. | $x_{min}$ | $x_{max}$ | $t_{x.max}$ | ${t_{x, max}/(x}_{max}-x_{min})$ |
| --- | --- | --- | --- | --- |
| 1 | 5.98 | 64.96 | 15.00 | 0.25 |
| 2 | 5.51 | 45.69 | 11.00 | 0.27 |
| 3 | 6.02 | 118.70 | 27.00 | 0.24 |
| 4 | 6.46 | 76.58 | 18.00 | 0.26 |
| 5 | 5.98 | 88.97 | 21.00 | 0.25 |
| 6 | 8.65 | 154.92 | 35.99 | 0.25 |
| 7 | 7.36 | 169.46 | 40.00 | 0.25 |
| 8 | 5.55 | 36.40 | 9.00 | 0.29 |
| 9 | 7.23 | 52.79 | 12.00 | 0.26 |
| 10 | 7.44 | 80.75 | 19.00 | 0.26 |

The result for line swiching test is same for different energy as the table shown below:

| Spot X distance (cm) | Model X-time (ms) | Spot Y distance (cm) | Model Y-time(ms) | Diagonal Scan time record by log file (ms) |
| --- | --- | --- | --- | --- |
| 5 | 11.3353 | 5 | 42.9314 | 41.9990 |
| 2.5 | 6.8842 | 2.5 | 21.8624 | 21.0000 |
| 10 | 24.2000 | 10 | 55.0655 | 53.9990 |
| 2.5 | 6.8842 | 5 | 42.9314 | 41.9990 |
| 5 | 11.3353 | 10 | 55.0655 | 53.9990 |
| 10 | 24.2000 | 20 | 82.7930 | 81.9990 |
| 5 | 11.3353 | 2.5 | 21.8624 | 20.9980 |
| 10 | 24.2000 | 5 | 42.9314 | 41.9980 |
| 20 | 50.2000 | 10 | 55.0655 | 55.0000 |
| 10 | 24.2000 | 2.5 | 21.8624 | 22.9900 |
| 20 | 50.2000 | 2.5 | 21.8624 | 43.9990 |
| 15 | 37.2000 | 2.5 | 21.8624 | 36.0000 |
| 10 | 24.2000 | 2 | 14.8745 | 23.0000 |
| 8 | 19.0000 | 2 | 14.8745 | 18.0000 |
| 9 | 21.6000 | 2 | 14.8745 | 21.0000 |
| 12 | 29.4000 | 2 | 14.8745 | 28.0000 |
| 13 | 32.0000 | 2 | 14.8745 | 30.0000 |
| 15 | 37.2561 | 3 | 28.2561 | 35.0000 |
| 8 | 19.0000 | 2 | 14.8745 | 19.0000 |

Table s5: The diagonal switching time ($t_{RSW}$) experiment result

From the 102 clinical IMPT beams log file, we obtain the following results.

Table s6: More information of the deviation comparison with log files.

|  | P1 model | IBA’s prediction |
| --- | --- | --- |
| Mean of relative difference in percentage | 2.06% | 58.00% |
| Absolute time of the mean difference(s) | 2.10 | 58.28 |
| Standard deviation of relative difference | 3.00% | 15.26% |
| Absolute time of the standard deviation (s) | 3.56 | 26.16 |
| Largest relative deviation in percentage | 9.28% | 99.52% |
| Absolute time of the largest deviation | 13.83 | 180.01 |

The current IBA ProteusONE®,’s treatment console (or called ScanAlgo), was not able to predict an accurate BDT (a brain case as an example).


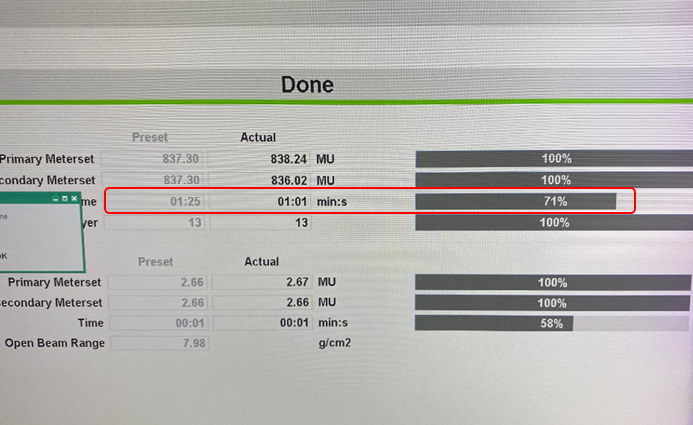


Figure s1: The control interface of the IBA ProteusONE® in Beaumont proton therapy center to predict the delivery time for single beam. Prediction time 29% error shows in this brain case as an example.

The uncertainly look-up table is recorded as stability excel spreadsheet in every log file :

Figure s2: A morning machine look-up table reflects the relationship between uncertainty and VDee(%).

The beam meterset calibration at our facility :


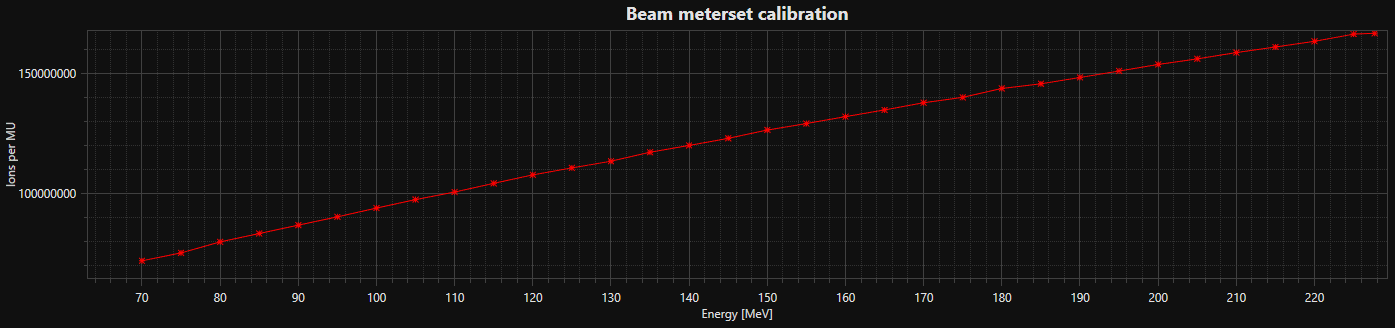


Figure s3: The relationship of ion numbers per MU.

The stability excel spreadsheet in every log file also records the following information:

Figure s4: A morning machine look-up table reflects the relationship between mean charge per pulse and VDee(%).

To model the burst switching time (BST), we analyzed the log files structure below.


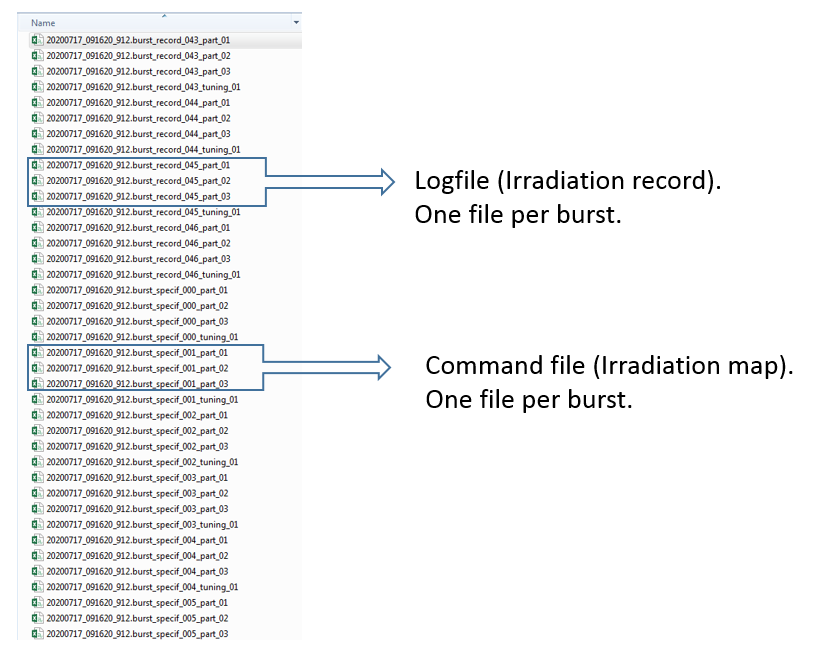


Figure s5: An example of the log file package from the IBA ProteusONE system. It includes a series of burst irradiation record files such as burst_record_045_part01 (1^st^ burst irradiation record of energy layer 45.) and burst_record_045_part02 (2^nd^ burst irradiation record of energy layer 45). It also includes a series of burst command files such as burst_specif_001_part01(1^st^ burst command file of energy layer 1.) and burst_specif_001_part02 (2^nd^ burst command file of energy layer 2). Burst switching time (BST) is related to these two types of files that communicates information between the workstation and proton delivery system.

To further understand the data saving structure for log record file,


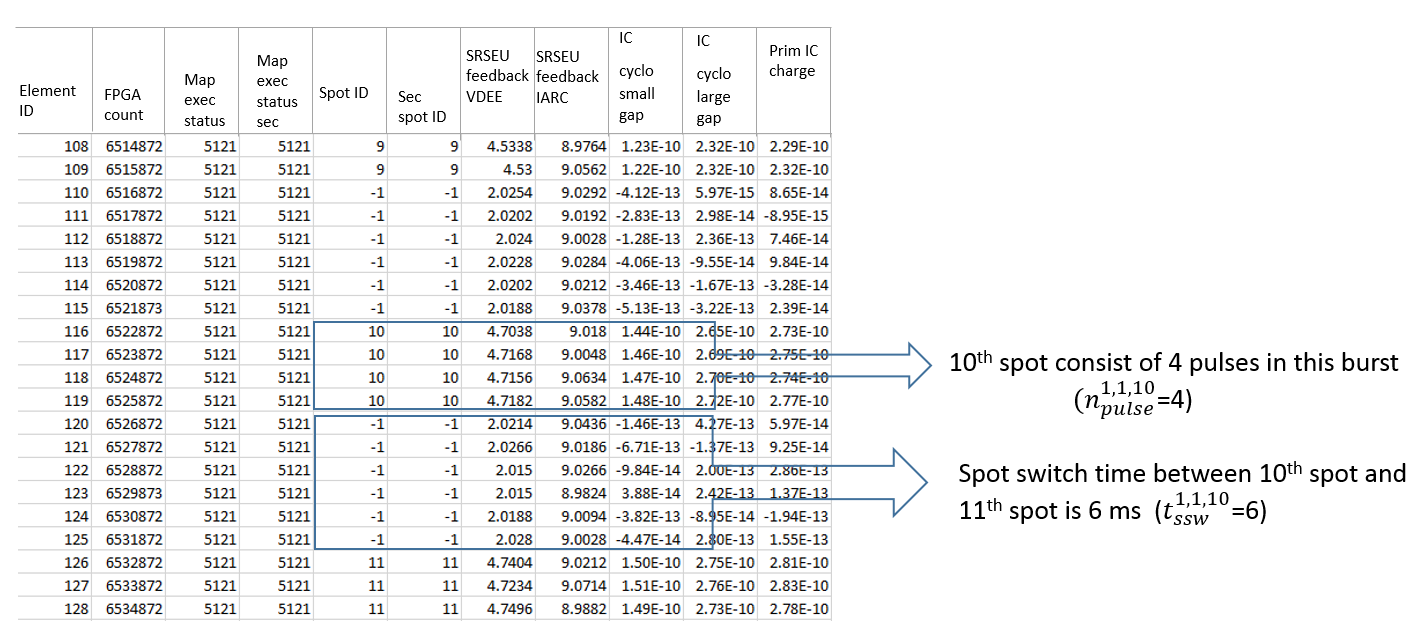


Figure s6: The proton system records the radiation delivery information in the csv file row by row at 1kHz (each row costs 1ms at a rate of 1kHz). This machine log file size reflects the irradiation number of the previous radiation burst ( ${\boldsymbol{N}_{\boldsymbol{SSW}}^{\boldsymbol{i,j}}\boldsymbol{+N}}_{\boldsymbol{pulse}}^{\boldsymbol{i,j}}$ ), which were obtained from the “Spot ID” column. The number “-1” in the excel represents this row records spot switching. The positive integers in the same column indicate the spot id containing pulses. In this example, the spot switching between 10^th^ and 11^th^ spot records 6 rows are needed for 6ms.

The same way to analyze the data saving structure of the command file,


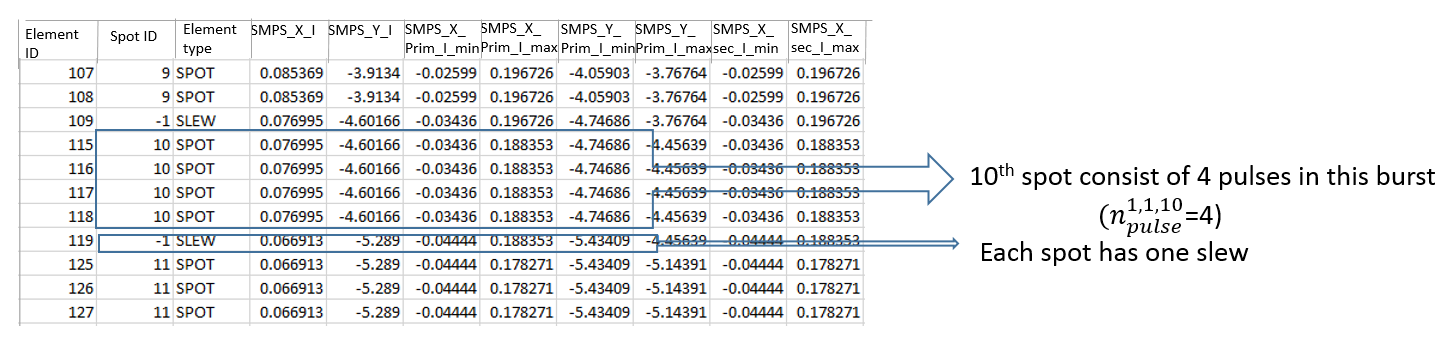


Figure s7: The command file size reflects the number of the pulse and spot to be delivered in the next burst ( $\boldsymbol{N}_{\boldsymbol{pulse}}^{\boldsymbol{i,j+1}}\boldsymbol{+}\boldsymbol{N}_{\boldsymbol{spot}}^{\boldsymbol{i,j+1}}$ ), which are obtained from the “Spot ID” column. The positive integers before the “SPOT” indicate the spot id containing pulses.

To evaluate the interplay effect, we created the following plan.


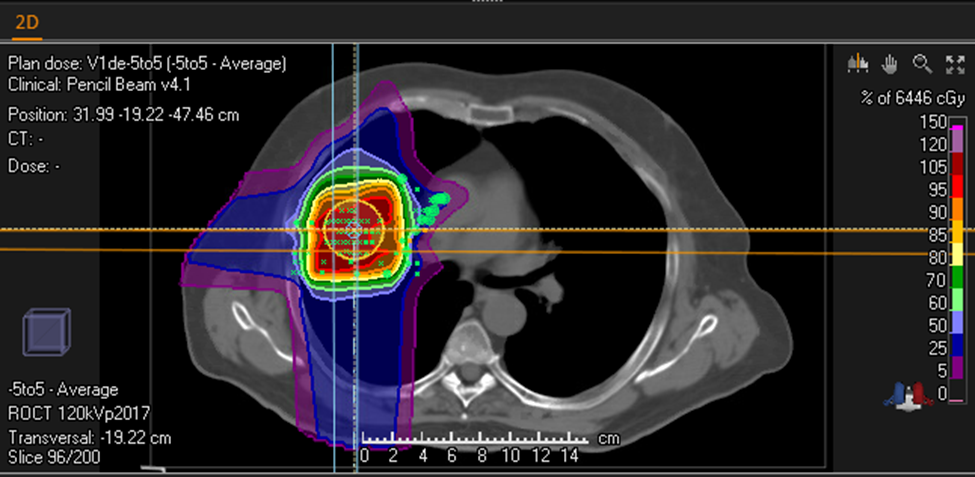


Figure s8: Two-field Single Field Uniform Dose (SFUD) plans were generated in treatment planning system.

Data transmission information between two bursts contains 4 parameters: the total spot switching number in last burst ( $\boldsymbol{N}_{\boldsymbol{SSW}}^{\boldsymbol{i,j}}$, unit in ms), the total number of pulse in last burst ( $\boldsymbol{N}_{\boldsymbol{pulse}}^{\boldsymbol{i,j}}$), the total number of pulse in next burst ( $\boldsymbol{N}_{\boldsymbol{pulse}}^{\boldsymbol{i,j+1}}$) and the total number of spot in next burst ($\boldsymbol{N}_{\boldsymbol{spot}}^{\boldsymbol{i,j+1}}$). We plot those information and log file size from 10 clinic IMPT beams.

Figure s9: The record log file size is proportional to ${\boldsymbol{N}_{\boldsymbol{SSW}}^{\boldsymbol{i,j}}\boldsymbol{+N}}_{\boldsymbol{pulse}}^{\boldsymbol{i,j}}$ .

Figure s10: The command file size is proportional to $\boldsymbol{N}_{\boldsymbol{pulse}}^{\boldsymbol{i,j+1}}\boldsymbol{+}\boldsymbol{N}_{\boldsymbol{spot}}^{\boldsymbol{i,j+1}}$ .


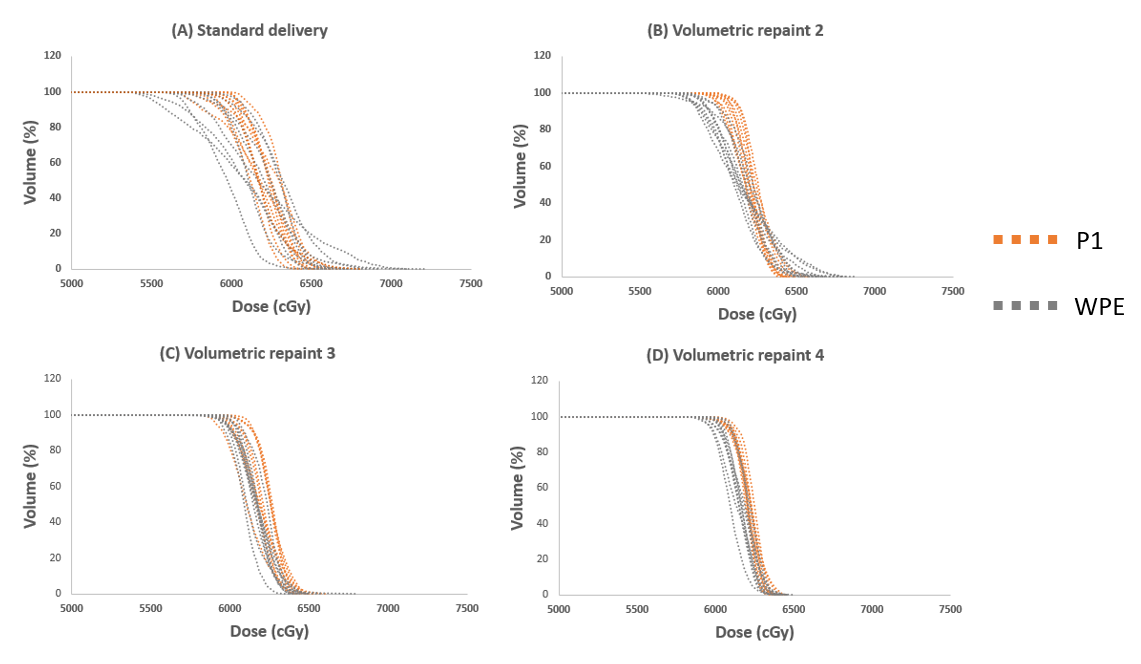


Figure s11: DVH band curve of interplay effect for CTV coverage comparison between machine-specific ProteusONE^®^ model (labeled by “P1”, a burst mechanism) and WPE model (a non-burst mechanism). Various dotted lines are for different starting phase.

To test if this in-house IBA ProteusONE® delivery time and sequence model could help better estimate the treatment irradiation time and daily patient treatment throughput, a total of 12 cases from the four disease sites such as prostate, head and neck, lung, and chest wall cancer were retrospectively selected. Beam delivery time was predicted by the in-house model and IBA’s treatment console (or called ScanAlgo). The actual irradiation time was obtained from the log files.


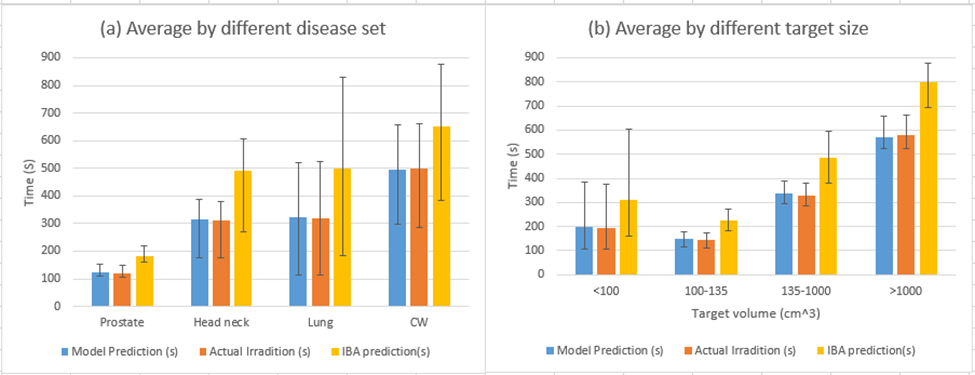


Figure s12: The model prediction time compared to the actual irradiation time in the clinical workflow.

To study how daily variation affect the model accuracy, we track a completed treatment head and neck case with 25 fractions across 38 days.


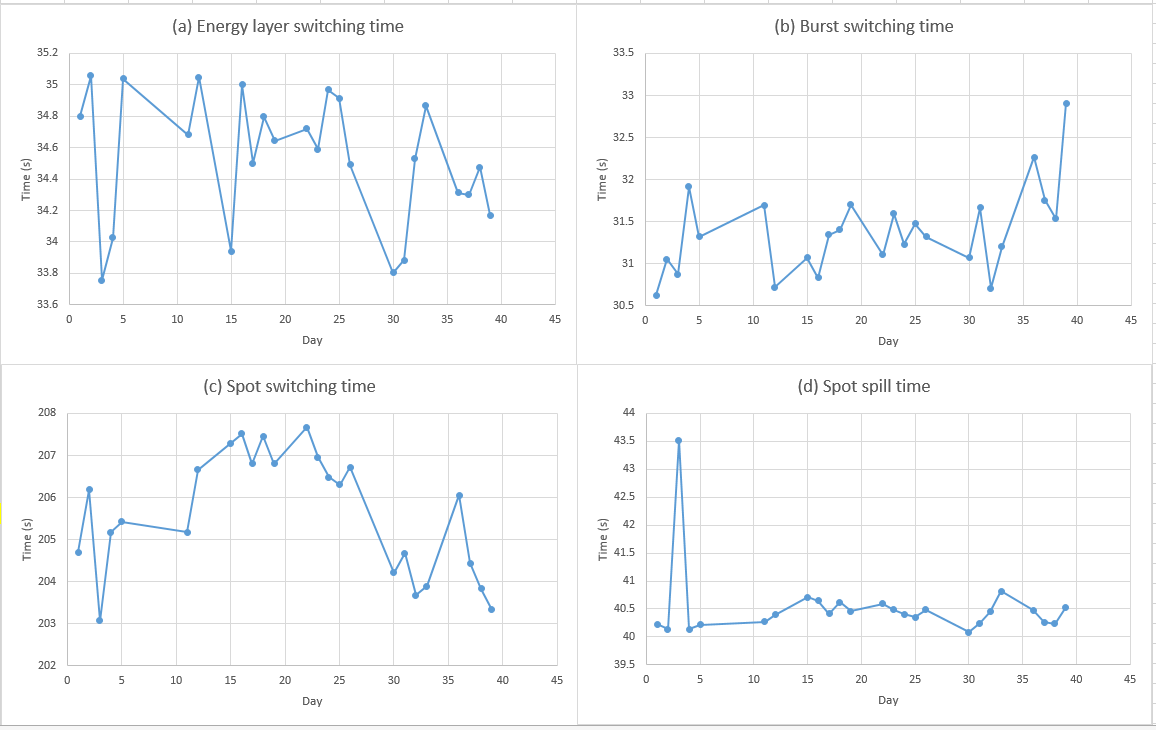


Figure s13: Log file recorded each time component changes in a 25 fractions treatment.


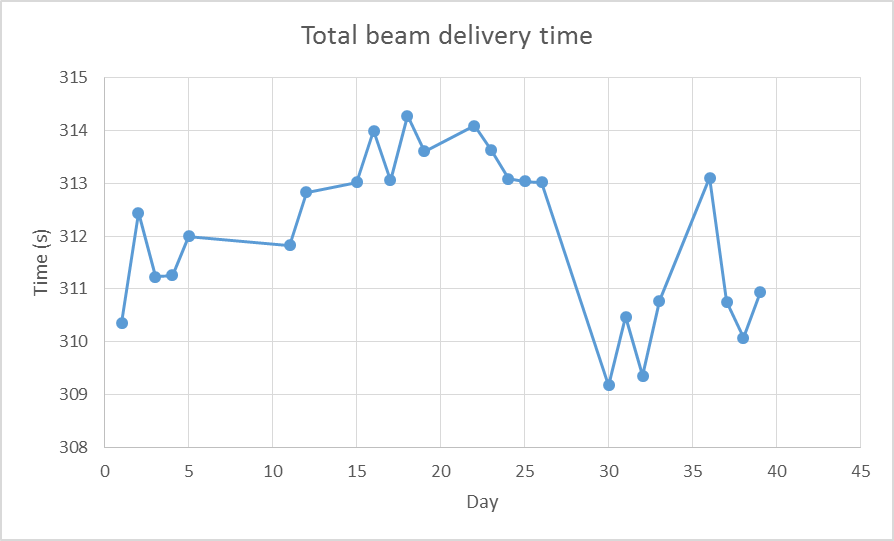


Figure s14: Log file recorded total BDT changes in a 25 fractions treatment.
